# CEUS of Focal Splenic Lesions: Enhancement Patterns and Differentiation of Benign and Malignant Disease

**DOI:** 10.3390/diagnostics16213621

**Published:** 2026-11-02

**Authors:** Kathleen Möller, Jan Nawratil, Christian Jenssen, Michael Gerber, Sebastian Zeissig, Christoph F. Dietrich

**Affiliations:** 1 Medical Department I/Gastroenterology, SANA Hospital Lichtenberg, 10365 Berlin, Germany; 2 Department of Visceral Surgery and Medicine, Inselspital, Bern University Hospital, University of Bern, 3010 Bern, Switzerland; 3 Department of Internal Medicine, Krankenhaus Märkisch Oderland, 16269 Wriezen, Germany; c.jenssen@khmol.de; 4 Brandenburg Institute for Clinical Ultrasound (BICUS), Brandenburg Medical University, 16816 Neuruppin, Germany; 5 Center of Excellence and Education, Haldenweg 11A, 3626 Thun, Switzerland

**Keywords:** spleen, splenic lesions, contrast-enhanced ultrasound, CEUS, hemangioma, lymphoma, metastases, washout pattern

## Abstract

Focal splenic lesions are uncommon, and diagnostic experience with splenic imaging is substantially more limited than that with focal liver lesions. Contrast-enhanced ultrasound (CEUS) has emerged as a valuable imaging modality for the detection and differentiation of splenic lesions because it allows real-time assessment of vascularization and enhancement behavior without ionizing radiation. This narrative review summarizes the current evidence regarding CEUS findings in focal splenic lesions, with particular emphasis on the differentiation between benign and malignant lesions. Benign vascular lesions typically demonstrate arterial hyperenhancement or isoenhancement with persistent enhancement or only mild late washout, whereas malignant lesions frequently show early and progressive washout with marked hypoenhancement in the parenchymal phase. Rapid washout, particularly within the first 60 s after contrast administration, represents one of the most important imaging features associated with malignancy. Although overlap of enhancement patterns exists and standardized diagnostic criteria are still lacking, CEUS represents a valuable, radiation-free, and cost-effective imaging modality for the evaluation of focal splenic lesions. CEUS may improve diagnostic confidence, support clinical decision-making and reduce the need for additional cross-sectional imaging in selected patients.

## 1. Introduction

Focal splenic lesions are uncommon, and diagnostic imaging experience with splenic disease is considerably more limited than that with focal liver lesions. In a multicenter study including 50,000 patients, fewer than 0.6% had a splenic lesion [1], while in a university ultrasound (US) department, splenic lesions were identified in only 0.06% of 285,000 examinations [2]. Consequently, both the learning curve and the level of diagnostic confidence for splenic imaging are substantially lower than for hepatic lesions. This also applies to contrast-enhanced ultrasound (CEUS), despite its increasing use in abdominal imaging.

Histological confirmation of splenic lesions remains more challenging than in the liver because of the potential risk of bleeding, although modern image-guided biopsy techniques have improved its safety profile.

Focal splenic lesions comprise a broad spectrum of entities, including benign and malignant tumors, inflammatory and granulomatous lesions, vascular emergencies such as infarction and pseudoaneurysm, cystic lesions, and traumatic injuries (Table 1). Most incidental splenic lesions are benign. Nevertheless, in a biopsy study, 38% of asymptomatic patients still had a lesion that the authors considered relevant [3]. In patients without a history of malignancy, more than 90% of splenic lesions are benign, and even in patients with known malignancy, most lesions remain non-malignant [2,4]. Nevertheless, differentiation between benign and malignant lesions is clinically important because splenic involvement may influence staging, prognosis, and therapeutic management.

The key diagnostic challenge is that B-mode ultrasound alone is insufficient for reliable lesion characterization. Both benign and malignant splenic lesions may demonstrate variable echogenicity and can appear hyperechoic, isoechoic, or hypoechoic. The value of color Doppler ultrasound in diagnosing focal splenic lesions is limited [5,6,7]. Additional imaging with contrast-enhanced computed tomography (CT) or magnetic resonance imaging (MRI) is frequently required [8,9,10,11]. However, CEUS has emerged as a valuable imaging modality for the characterization of focal lesions in multiple organ systems because it enables real-time evaluation of vascularization and enhancement behavior without ionizing radiation [12,13].

In the spleen, CEUS offers several potential advantages. It improves lesion detection compared with native B-mode ultrasound, allows dynamic assessment of arterial and parenchymal enhancement patterns, and may support differentiation between benign and malignant lesions. Certain enhancement features, particularly the presence, timing, and degree of washout, appear to be diagnostically relevant. In addition, CEUS is increasingly used for the evaluation of traumatic, ischemic, inflammatory, and infectious splenic lesions, especially in patients requiring radiation-free follow-up imaging.

Despite the growing use of CEUS in splenic imaging, the available literature remains limited and heterogeneous. Most published studies are retrospective single-center series or case reports, and standardized diagnostic criteria for splenic CEUS have not yet been established. Furthermore, enhancement patterns may overlap between different entities, making interpretation challenging in clinical practice.

The primary aim of this review is to summarize CEUS enhancement patterns of focal splenic lesions and to evaluate the diagnostic contribution of CEUS for differentiating benign from malignant splenic disease. A detailed description of infectious, ischemic, and traumatic lesions is not provided in this review; however, sarcoidosis is included because of its importance in the differential diagnosis of solid focal splenic lesions.

## 2. Methods

### Search Strategy

**Figure 1:**
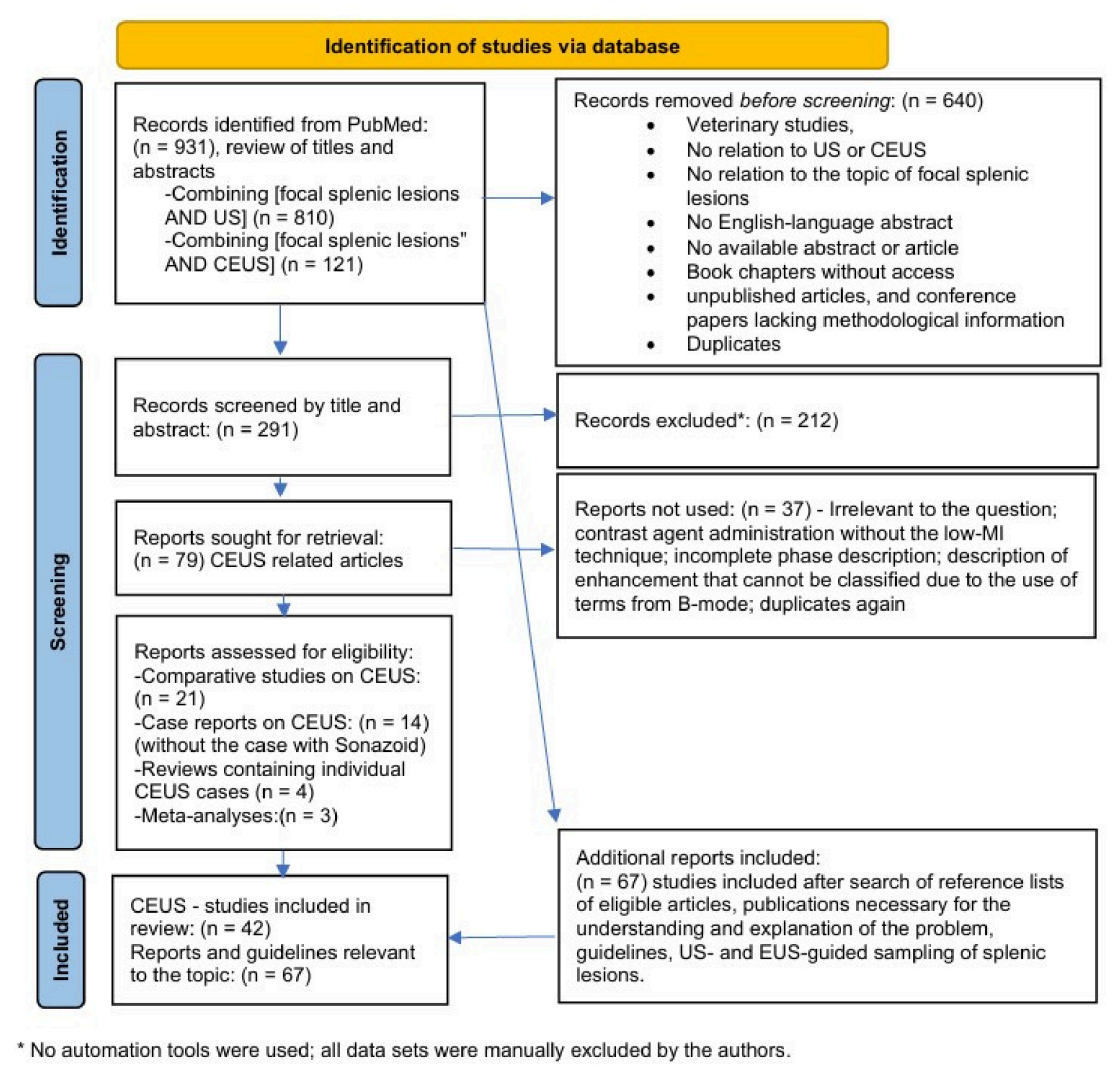
Literature search and study selection process. The flow diagram is based on the PRISMA 2020 framework and was adapted to the structured literature search used for this narrative review.

This narrative review was informed by a structured literature search of PubMed. The search covered the period from 1990 to 31 March 2026, with an updated search for CEUS-related publications completed on 20 September 2026. The literature search and study selection process are summarized in Figure 1. The ultrasound contrast agent SonoVue (SonoVue, Bracco, Milan, Italy) has been approved since March 2001; therefore, studies on SonoVue were not expected until after this date. Additional studies from reference lists dating from before 1990 were included if they were beneficial to understanding the lesions. There was no time limit on conducting targeted follow-up research to better understand the lesions, the pathology, and statistical data over the course of the review.

The literature search focused on CEUS enhancement of various focal splenic lesions and differentiation between benign and malignant splenic lesions. A double independent review procedure was performed (K.M., C.J.). Any ambiguities or disagreements were resolved through discussion. The results of the literature review were then critically reviewed by all co-authors and discussed in cases where opinions differed. The following keywords were used (the results are shown in parentheses): benign splenic lesions AND CEUS (23); malignant splenic lesions AND CEUS (38); splenic hemangioma AND ultrasound (329); splenic hemangioma AND CEUS (9); splenic hamartoma AND ultrasound (114); splenic hamartoma AND CEUS (4); splenic lymphangioma AND ultrasound (8); splenic lymphangioma AND CEUS (3); splenic littoral cell angioma AND ultrasound (83); splenic littoral cell angioma AND CEUS (2); sclerosing angiomatoid nodular tumor AND ultrasound (73); sclerosing angiomatoid nodular tumor AND CEUS (2); splenic sarcoidosis AND CEUS (7); splenic lymphoma AND CEUS (22); splenic metastases AND CEUS (8); splenic angiosarcoma AND ultrasound (203); splenic angiosarcoma AND CEUS (3).

The review primarily focused on studies using the ultrasound contrast agent SonoVue (Bracco, Milan, Italy), with one additional case report using Sonazoid (GE Healthcare AS, Oslo, Norway; Daiichi-Sankyo) included for its relevant description of the enhancement pattern [14]. This refers to the very rare sclerosing angiomatoid nodular transformation, in which the spoke wheel-like enhancement in the arterial phase is very well documented. The focus of the review was on analyzing the contrast enhancement of lesions in the arterial and parenchymal phases. Descriptions of some rare tumors were extracted from studies. For this purpose, the figures and figure legends were analyzed. If only an “enhancement” was described, without specifying the intensity relative to the surrounding splenic parenchyma, the data were not used. Most of the reviewed publications were in English-language studies. Relevant non-English-language articles were assessed using translation tools: DeepL (Deep Learning, Cologne, Germany) and Google Translate (Google, Mountain View, CA, USA). Articles without available abstracts were excluded unless the full publication was available. Veterinary studies, unpublished articles, and conference papers lacking methodological information were also excluded. Pediatric studies were included, particularly those focusing on solid splenic lesions. No automated screening or deduplication tools were used; record screening and duplicate identification were performed manually by the authors. The publications were sorted by author’s name and year of publication, which made it possible to identify duplicates.

Eligible publications included studies on CEUS of benign and malignant focal splenic lesions and their differential diagnosis; relevant studies on B-mode ultrasound and color Doppler imaging; guidelines addressing CEUS of the spleen; comparative studies, case reports, meta-analyses, and reviews providing sufficiently detailed descriptions of enhancement patterns; and publications addressing US- or EUS-guided tissue sampling of splenic lesions (Figure 1). Following screening and eligibility assessment, 42 CEUS-related publications were included in the core evidence synthesis, comprising 21 comparative studies, 14 case reports, 4 reviews containing individual CEUS cases, and 3 meta-analyses.

The case report with Sonazoid is included in the additional publications [14]. An additional 67 publications were identified through reference-list searches and targeted searches and were included to provide relevant background information on lesion pathology, diagnostic criteria, guidelines, and US- or EUS-guided tissue sampling. Thus, a total of 109 publications were considered in the review (Figure 1).

## 3. Imaging of Splenic Tissue in CEUS

The spleen consists of two different types of tissue: the red pulp and the white pulp. The red pulp contains four types of vascular structures: thin, non-anastomosing arterial vessels, thin-walled venous vessels (splenic sinusoids), cell plates (splenic cords) located between the sinusoids, and veins that drain the sinusoids. The white pulp is composed of lymphatic tissue [15]. The velocity of blood flow differs between the red and white pulp. This is important for the visualization of spleen tissue in imaging procedures using contrast agents.

Unlike the liver, the spleen has no dual arterial and portal venous blood supply. On CEUS with the ultrasound contrast agent (UCA) SonoVue^®^ (Bracco, Milano, Italy), two phases are distinguished: an arterial phase and a parenchymal phase. The arterial phase starts approximately 10–20 s after contrast agent administration and continues for about 35–40 s. The parenchymal phase begins approximately 30 to 45 s after contrast administration [13]. A very distinctive enhancement pattern is the unique heterogeneous enhancement of the spleen during the arterial phase in the first 30 s following UCA administration. This is also known as the “zebra effect” in contrast-enhanced imaging and is caused by temporal differences in blood flow into the red (earlier) and white pulp (later) [13].

The inhomogeneous contrast enhancement observed with splenic CEUS lasts for about 30 s and then transitions to uniform, homogeneous hyperenhancement within 60 s after UCA injection. A sustained, homogeneous enhancement lasting 5 min or longer is another characteristic feature of spleen tissue [13,16].

Lim et al. [17] reported that unlike in the liver and kidneys, the intensity of enhancement in the spleen does not decrease and is still detectable even after 5 min [17].

To describe the intensity of enhancement in focal lesions relative to the surrounding spleen tissue, the terms hyperenhanced, isoenhanced, hypoenhanced, and non-enhanced are used. Washout refers to a temporal reduction in lesion enhancement relative to the surrounding splenic parenchyma, resulting in hypoenhancement after initial enhancement. Persistent hypoenhancement from the arterial phase onward should not be classified as washout [12,13,18]. A washout can occur rapidly or slowly. In this context, von Herbay et al. [19] assessed the parenchymal phase at 60 s and later at 120–180 s. The washout can be moderate or marked.

### Performance of CEUS Compared to Native B-Mode US and Radiological Imaging Techniques

Contrast-enhanced ultrasound (CEUS) has a higher sensitivity than B-mode ultrasound for detecting splenic lesions. In a meta-analysis of eight studies, the sensitivity and specificity of the pooled CEUS results were 0.95 (95% confidence interval [CI]: 0.92–0.97) and 0.97 (95% CI: 0.90–0.99), respectively (I^2^ = 27.4%; area under the curve [AUC] of the pooled ROC curve = 0.97). The sensitivity and specificity of the pooled native ultrasound results were 0.70 (95% CI: 0.56–0.80) and 0.96 (95% CI: 0.76–0.99; I^2^ = 83.4%; AUC = 0.84), respectively [20]. A lower negative likelihood ratio indicated a better ability to rule out disease.

In the population evaluated in the cited meta-analysis, the estimated probability of a splenic lesion following a negative examination was lower for CEUS than for B-mode ultrasound; however, these post-test probabilities depend on the underlying disease prevalence [20].

A meta-analysis of 28 included studies analyzed the diagnostic effectiveness of various imaging techniques for differentiating between malignant and benign splenic lesions [21]. PET showed a pooled sensitivity of 93% and a specificity of 82.8%; computed tomography (CT) a sensitivity of 73.6% and a specificity of 89.2%; contrast-enhanced magnetic resonance imaging (CE-MRI) a sensitivity of 81.4% and a specificity of 94.2%; and CEUS a sensitivity of 91.1% and a specificity of 85.7%. In terms of AUC values, PET led with an impressive 92.0%, followed by CEUS with 91.4%, CT with 88%, and CE-MRI with 85.3%. The differences between the contrast-enhanced modalities were not statistically significant.

Although the reported pooled AUC values were numerically similar across some modalities, these estimates were derived from heterogeneous study populations, clinical indications, reference standards, and disease prevalences and therefore should not be interpreted as evidence of diagnostic equivalence or interchangeability [21].

In addition, substantial selection bias must be considered when comparing the available evidence across imaging modalities. Many CEUS studies include incidental or otherwise selected cohorts, whereas PET and MRI studies predominantly involve high-risk oncological populations.

When CEUS was routinely included in the evaluation of focal splenic lesions in patients with suspected hematologic/lymphoproliferative disorders using structured CEUS-based diagnostics, the use of advanced imaging was lower compared to another group undergoing CT, MRI, and PET (CT: 46% vs. 100%, *p* < 0.001; PET: 4% vs. 20%, *p* = 0.018). In distinguishing between hematologic/lymphoproliferative and non-hematologic lesions, CEUS demonstrated high sensitivity (90.9%) and good specificity (79.5%), while the integrated clinical, laboratory, and CEUS-based diagnostic tool improved specificity (94.9%) while maintaining good sensitivity (81.8%) [22].

In a meta-analysis of five primary diagnostic studies on CEUS, sensitivity ranged from 90% to 98% and specificity from 85% to 95% [23].

It should also be noted here that acoustic radiation force impulse (ARFI) elastography of focal splenic lesions was not reliable in distinguishing between benign and malignant splenic lesions [24]. Similarly, it was not possible to distinguish between benign and malignant causes of splenomegaly (with malignant infiltration) based on the differences in stiffness observed by ARFI elastography [25].

## 4. Benign Tumors

### 4.1. Vascular Tumors

Splenic hemangiomas are benign vascular tumors composed of endothelial-lined capillary or cavernous vascular channels. Hamartomas consist of an abnormal mixture of normal spleen tissue components [15]. However, there are no reliable vascular patterns that allow for the classification of the different lesions.

In a study on the differentiation of splenic lesions, Stang et al. [26] described 36 benign hemangioma/hamartoma-type lesions that exhibited homogeneous isoenhancement in both the arterial and parenchymal phases. In the arterial phase, two of these lesions showed transient hyperenhancement, and at the end of the parenchymal phase (without a specified time), eight lesions developed slight hypoenhancement compared to the surrounding parenchyma [26]. Görg et al. described benign vascular tumors that exhibit hyperenhancement in the arterial phase with variable contrast enhancement patterns and hypoenhancement in the parenchymal phase [27]. Due to the overlapping CEUS characteristics of hemangiomas and splenic hamartomas, many studies—and, sensibly, clinical practice as well—describe hemangiomas and hamartomas collectively as “benign vascular splenic tumors.”

### 4.2. Hemangioma

Hemangiomas are the most common benign tumors of spleen. Autopsy studies have described the prevalence of splenic hemangiomas as 0.3–14% [15]. Histopathologically, they appear as non-encapsulated proliferations of vascular channels of varying sizes, ranging from capillary to cavernous. These are blood-filled spaces separated from one another by thin fibrous septa or splenic pulp tissue. Splenic hemangiomas are usually cavernous and may present as single or multiple lesions [15]. Hemangiomas are usually small, solid lesions. Cavernous hemangiomas can contain both solid and cystic components [15].

In B-mode ultrasound, splenic hemangiomas can appear hyperechoic or hypoechoic. Color Doppler ultrasound usually does not show visible vessels [28]. In most cases, hemangiomas exhibit hyperenhancement or isoenhancement in the arterial phase [4,19]. The hyperenhancement may be ring-shaped, globular, or homogeneous. During the arterial and parenchymal phases, a centripetal enhancement can be observed. After 60 s, enhancement characteristics vary from hyperenhancement to moderate hypoenhancement. After 120–180 s, the majority of hemangiomas will show mild to moderate hypoenhancement [Table 2].

In the study of 75 benign and malignant splenic lesions, 52% of 23 hemangiomas showed peripheral nodular (globular) enhancement in the arterial phase [29]. This peripheral globular enhancement has been observed in focal splenic lesions exclusively in cases of hemangiomas [29]. However, the enhancement pattern of splenic hemangiomas is not as typical as that seen in the liver. While hemangiomas with isoenhancement and hypoenhancement in the arterial phase and washout in the parenchymal phase have also been observed in the spleen [29], this is atypical for liver hemangiomas [30,31,32] (Table 2).

**Table 2:** Case series describing ultrasound contrast- enhancement patterns in splenic hemangiomas.

Study	Patients	B-Mode US	Arterial Phase	Parenchymal Phase	Comments
Von Herbay 2009 [19]	n = 3	n.i.	Hyperenhancement: hypervascularization at the edges of the lesions in the early phase n = 3/3; Peripheral–globular–nodular pattern n = 2/3; Homogeneous hyperenhancement n = 1/3.	*At 60 s:* Isoenhancement n = 1. Hyperenhancement n = 1. Moderate hypoenhancement n = 1. *At 120–180 s:* Isoenhancement n = 1. Moderate hypoenhancement n = 2.	
Chiavaroli 2011 [33]	n = 5	n.i.	Hyperenhancement n = 2; Isoenhancement n = 3; Centripetal fill-in n = 2/5.	Isoenhancement n = 5 (100%).	
Yu 2012 [29]	n = 23	Hyperechoic 20/23 Isoechoic 1/23 Hypoechoic 2/23	Hyperenhancement 16/23; Isoenhancement 3/23; Hypoenhancement 4/23.	Hyperenhancement 18/23. Hypoenhancement 5/23.	Peripheral nodular (globular) enhancement in AP in 52%.
Taibbi 2012 [28]	n = 27	Homogeneous 77.8% (mostly hyperechoic); inhomogeneous 22.2%; no CDI signals in 81.5%.	Different enhancement in AP and isoenhancement in PP (59.2%); Among these, 7.2% had globular enhancements followed by a centripetal fill-in.		
			Hypoenhancement in AP, hypoenhancement in PP (29.6%).		
			Hyper-/or isoenhancement in AP and hypoenhancement in late PP (11.1%).		
Li 2014 [34]	n = 7	Hyperechoic 42.9%; Hypoechoic 42.9%; Mixed echogenicity 14.3%.	Homogeneous/or heterogeneous hyperenhancement in AP and hyper-/or isoenhancement in PP (28.6%).		
			Isoenhancement in AP and slow washout in PP (14.3%).		
			Hypoenhancement in AP and PP (57.2%).		
Picardi 2022 [4]	Capillary hemangiomas n = 10	n.i.	Isoenhancement.	Hypoenhancement without subsequent washout.	Study with spleen nodules in patients with NHL or HL.
	Cavernous hemangiomas n = 10	n.i.	Discontinuous globular peripheral border.	Progressive centripetal enhancement or a very delayed (>3 min) mild washout.	

n.i. = no information available; AP = arterial phase; PP = parenchymal phase; CDI = color Doppler imaging; s = seconds.

### 4.3. Hamartoma (Splenoma/Nodular Hyperplasia)

A splenic hamartoma is a malformation consisting of an abnormal mixture of normal red splenic pulp elements. Autopsy studies have described the prevalence of splenic hamartomas as 0.024% to 0.13% [15].

**Figure 2:**
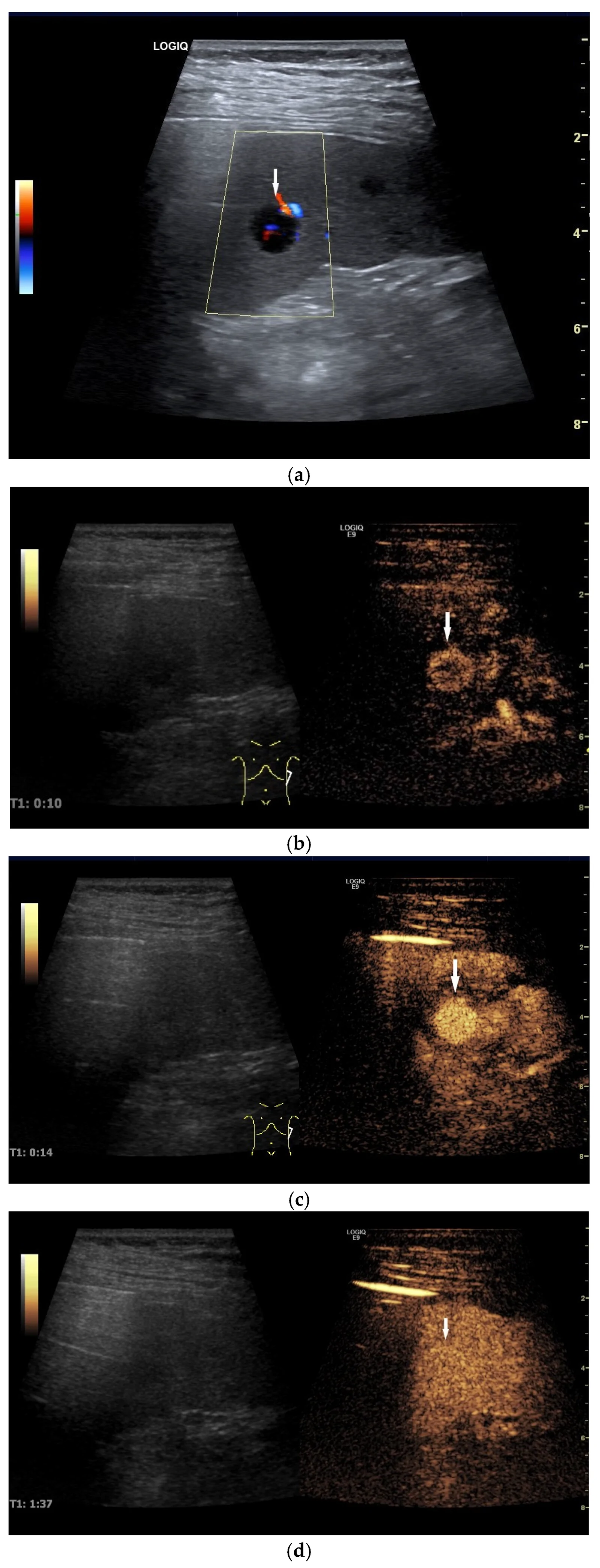
Benign vascular tumor. Incidental finding in a patient with elevated renal retention parameters. Hypoechoic splenic lesion measuring 12 mm with afferent and efferent vessels at the periphery and a central vessel on CDI (indicated below with arrows) (**a**). In the early arterial phase, the lesion shows peripheral contrast enhancement (**b**). Centripetal enhancement with homogeneous hyperenhancement develops very rapidly at 14 s (**c**). In the parenchymal phase, the lesion is isoenhanced at 1:37 min (**d**). Isoenhancement persisted over the parenchymal phase. The lesion was assessed as a benign vascular lesion without the need for further diagnostic testing. A follow-up examination was performed after 6 months without any change in findings.

**Figure 3:**
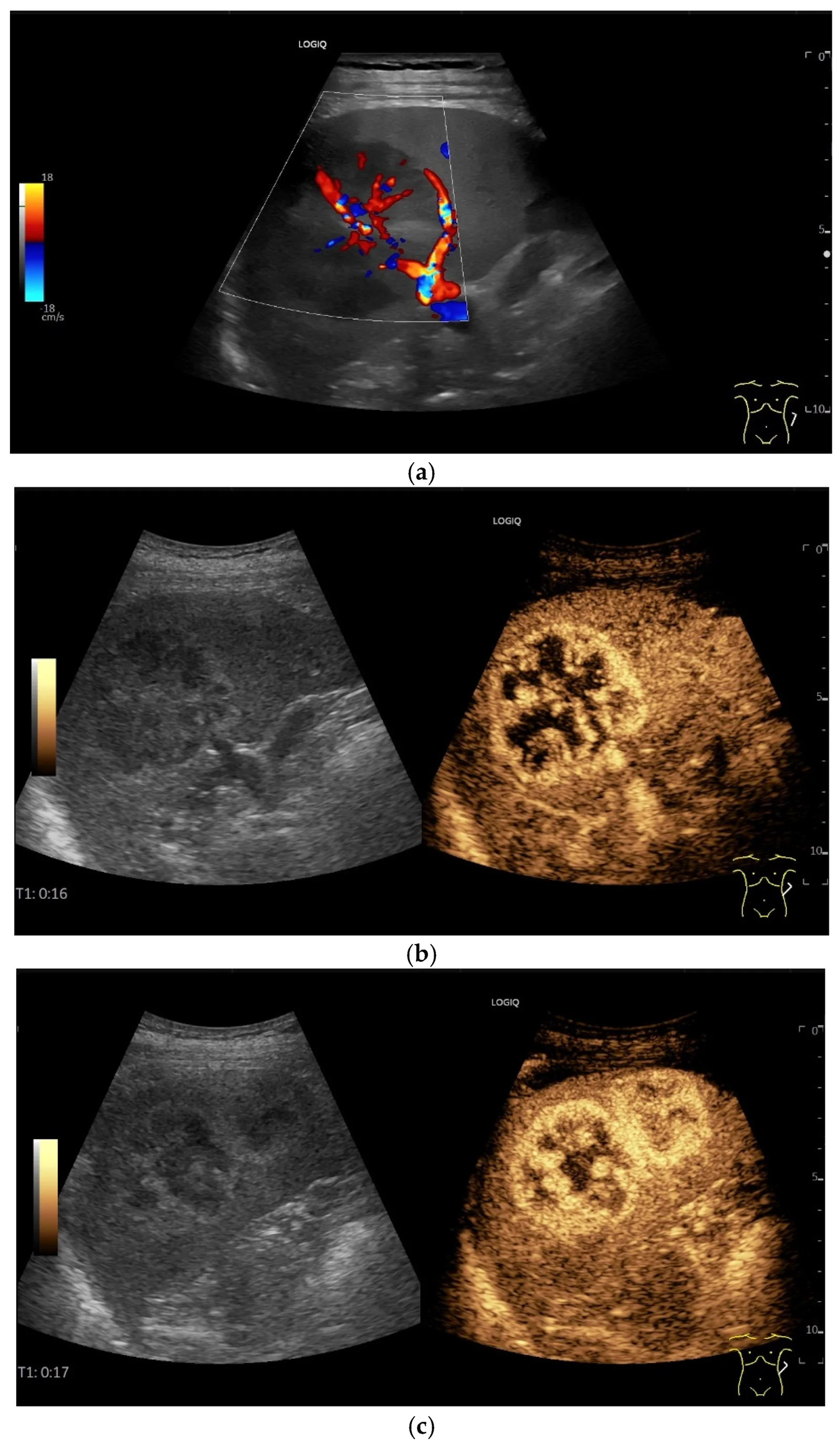

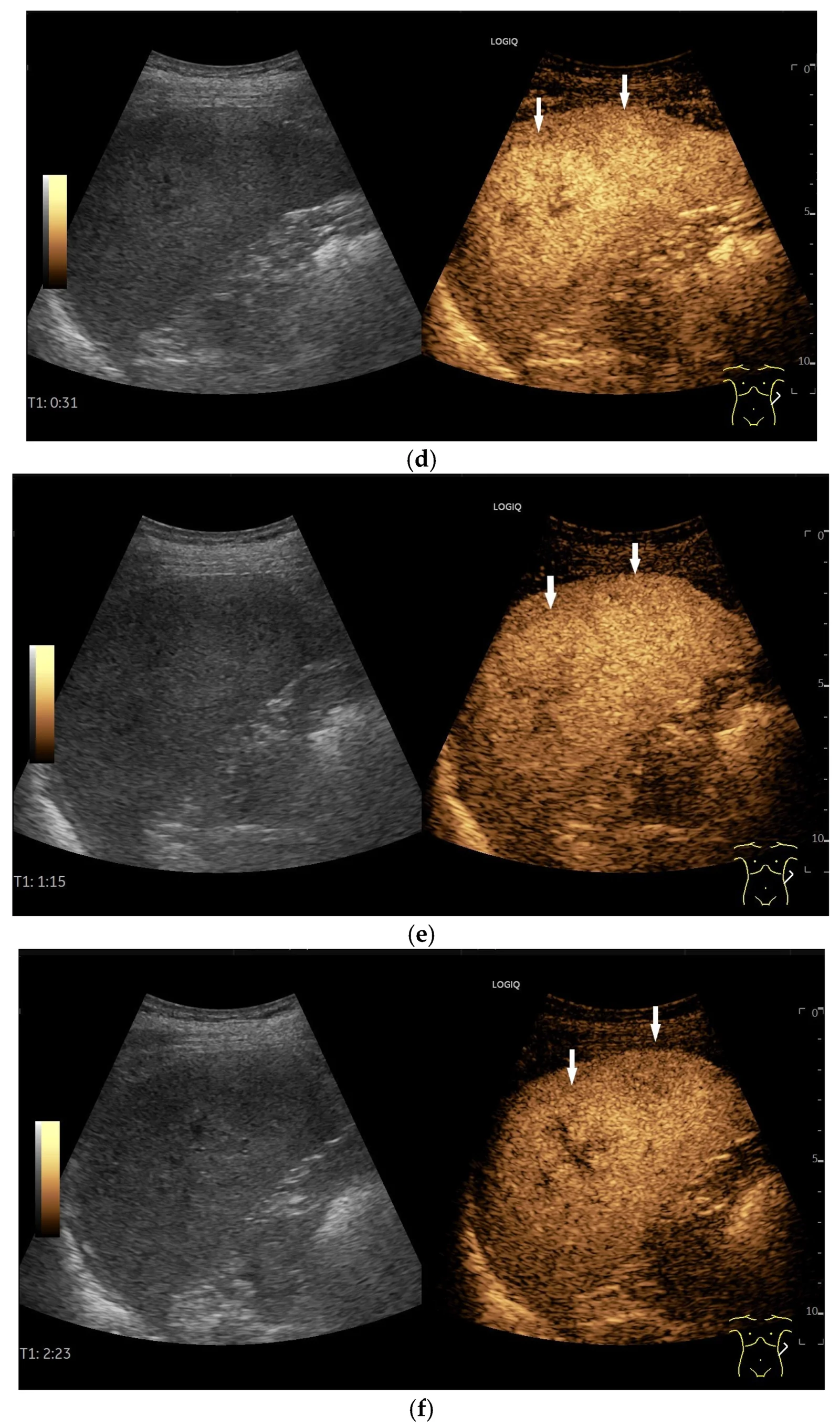
Benign vascular tumor. Incidental finding in a patient with sigmoid diverticulitis. Hypoechoic lesion with a central hyperechoic reflex and, on CDI, spoke-like vascular bifurcation (**a**). CEUS shows ring-shaped enhancement with globular contrast enhancement at the periphery (**b**). Over time, the globular contrast lakes enlarge. A second, similar lesion is visible adjacent to the lesion (**c**). At the end of the arterial phase, the lesion is almost completely hyperenhanced except for a small central hypoenhanced area (**d**). During the parenchymal phase at 1:15 min (**e**) and 2:23 min (**f**), the lesion (arrow) remains slightly hyperenhanced except for the central area, which shows faint hypoenhancement. The lesions were assessed as benign vascular tumors.

**Figure 4:**
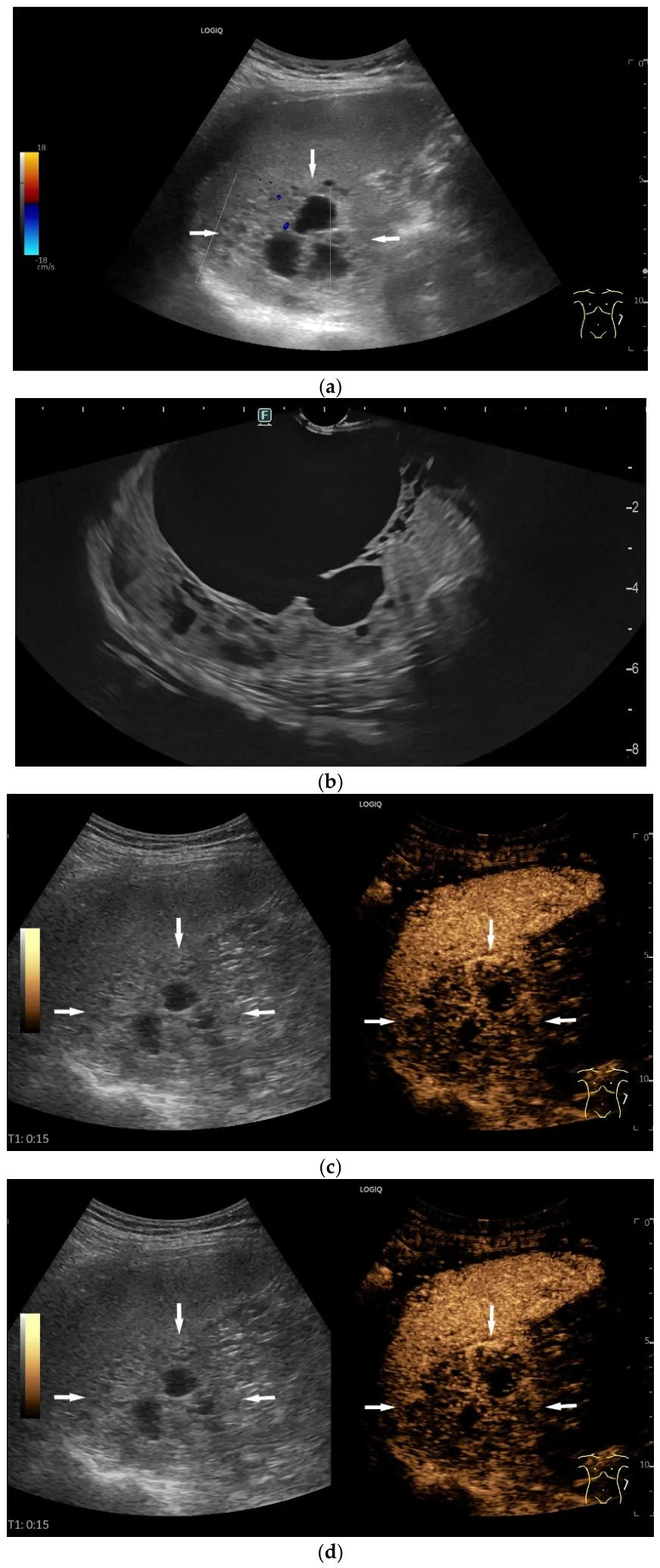

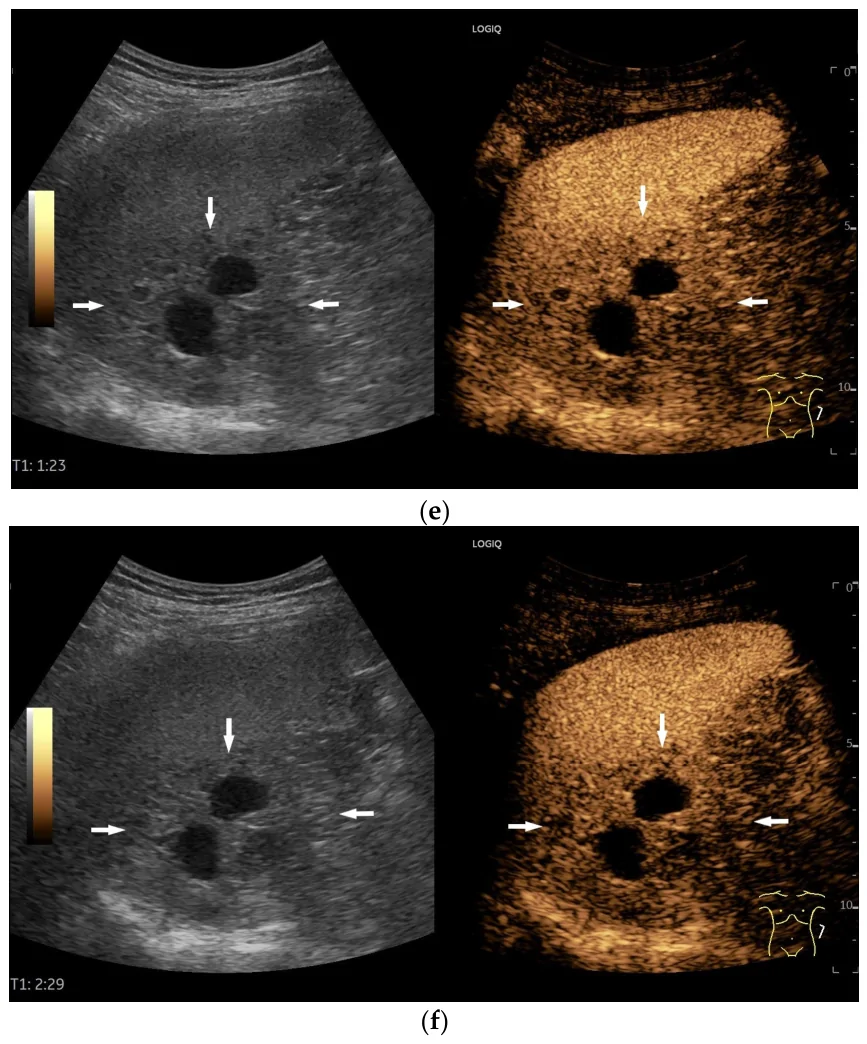
Cavernous hemangioma. Complaints in the upper left abdomen. A 60 mm cystic–solid mass (indicated below with arrows) is located adjacent to the splenic parenchyma of the upper third of the spleen. The solid components are isoechoic, slightly hyperechoic compared to the spleen. The lesion cannot be definitively assigned to the spleen (**a**). Therefore, MRI and EUS (**b**) are performed. The lesion is assigned to the spleen, not to the tail of the pancreas or the left adrenal gland. On MRI, the lesion’s assignment remains unclear. On CEUS, the lesion shows enhancement later than the splenic tissue (**c**). In the arterial phase, the solid components remain slightly hypoenhanced compared to the splenic tissue (**d**). The solid components of the lesion remain slightly hypoenhanced in the parenchymal phase at 1:23 min and 2:29 min (**e**,**f**), while the cystic components are non-enhanced. No further washout occurred. The lesion was assessed as non-malignant but could not be definitively assigned. The patient requested a splenectomy; histology revealed cavernous hemangioma.

**Figure 5:**
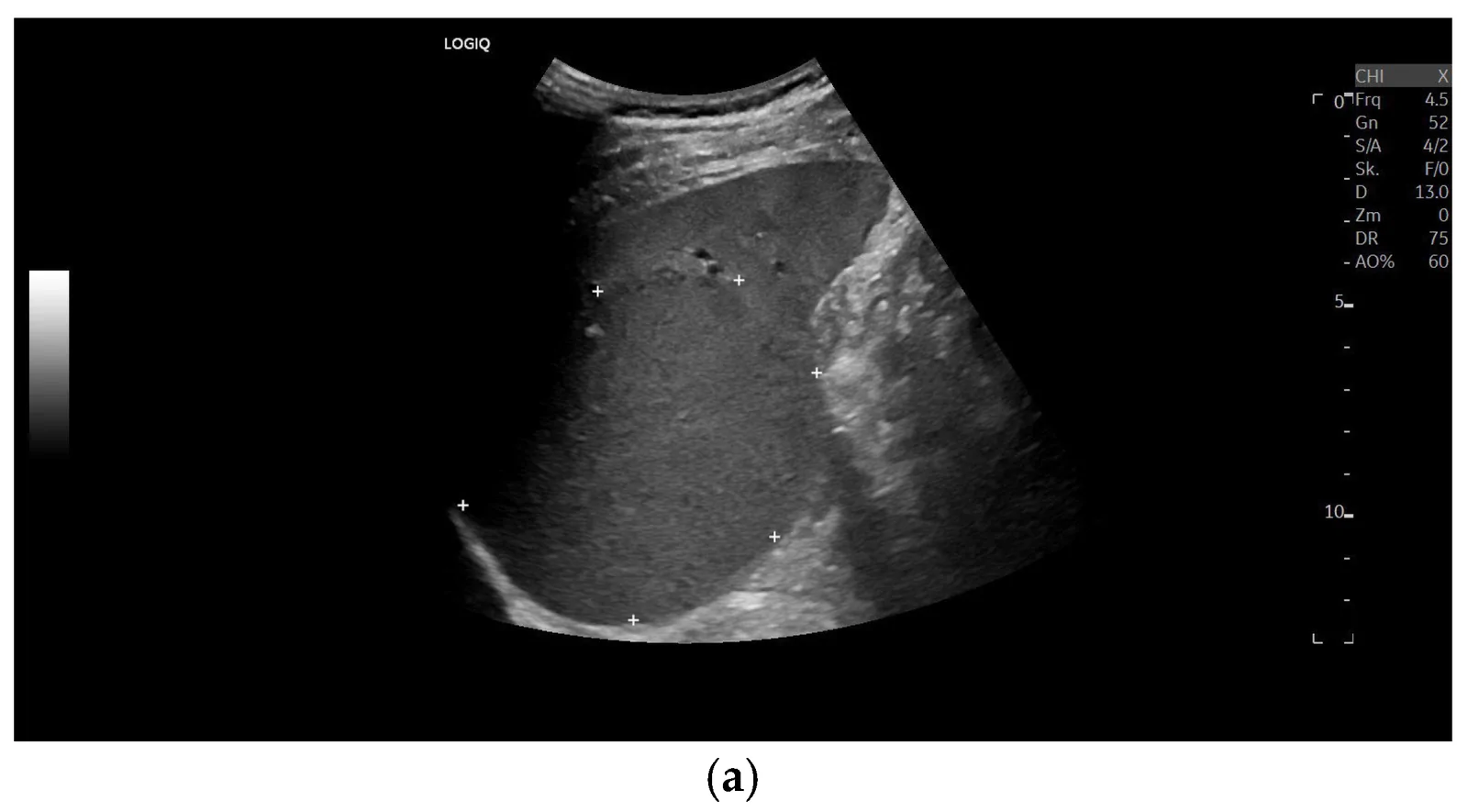

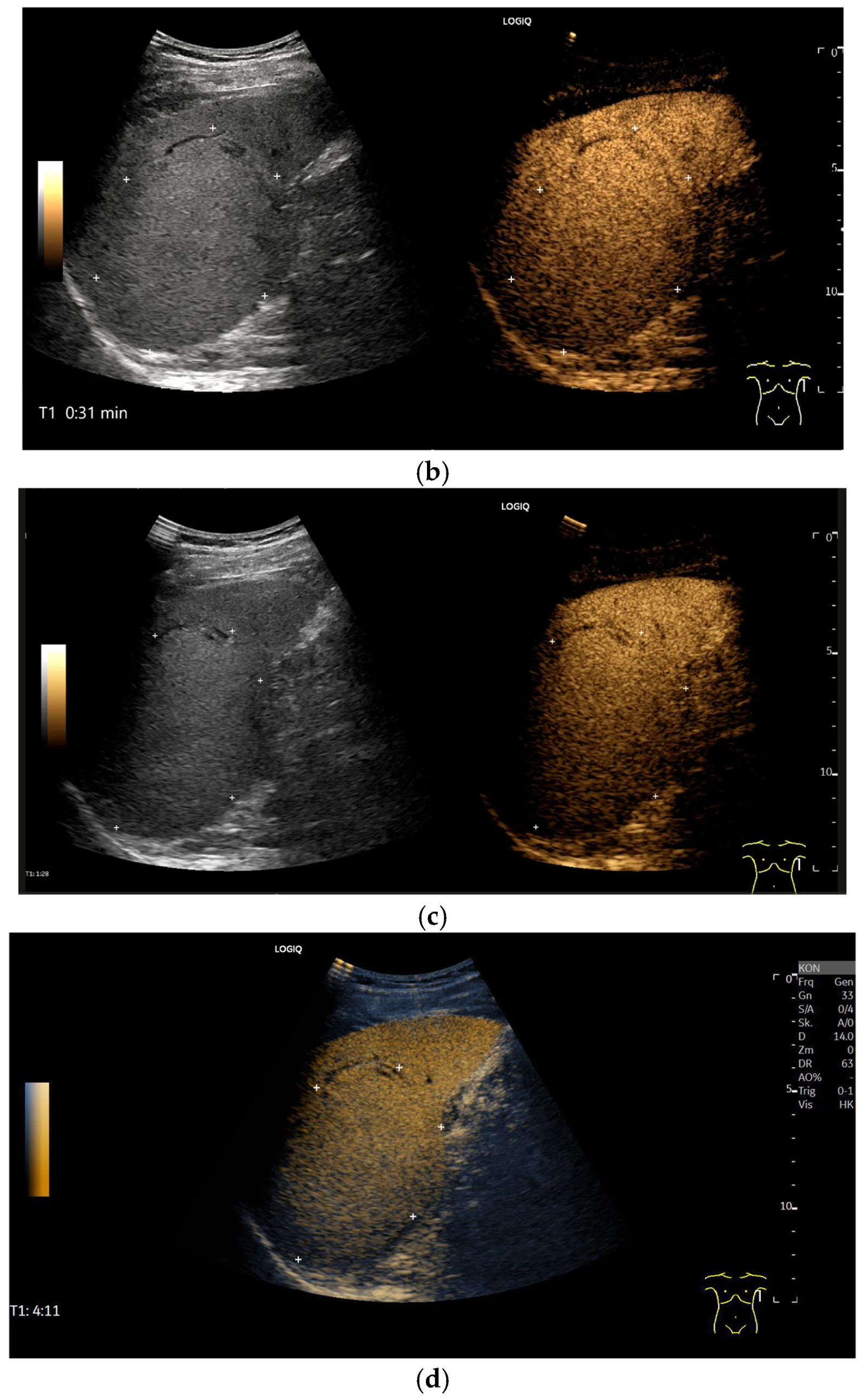
Hamartoma. The patient presented with recurrent left-sided flank pain and a well-defined isoechoic to minimally hypoechoic splenic lesion on ultrasound (between the markers) (**a**). In the arterial phase (0:31 min), this lesion was isoenhanced (**b**). The isoenhancement remained detectable in the parenchymal phase at 1:28 min (**c**) and at 4:31 min (**d**). The lesion was assessed as a benign vascular splenic lesion. Since the patient continued to experience recurrent left-sided flank pain and, in the meantime, also showed a small amount of free perisplenic fluid with fibrin around the spleen, a splenectomy was performed. Histology revealed hamartoma.

Hamartoma is a solid homogeneous mass with varied echogenicity relative to the normal splenic parenchyma, with evidence of vessels on color Doppler imaging (CDI) [15,35,36,37]. Stang et al. describe benign vascular tumors of the hemangioma/hamartoma type, but they describe no explicit enhancement pattern on CEUS specifically for hamartomas [38]. Table 3 contains enhancement patterns extracted from individual descriptions from studies. Various benign vascular tumors of the spleen are shown in Figure 2, Figure 3, Figure 4 and Figure 5.

### 4.4. Lymphangioma

Although splenic lymphangiomas are frequently cystic rather than truly solid lesions, they are included because they represent an important differential diagnosis of focal splenic abnormalities encountered during ultrasound examination. Lymphangiomas are caused by abnormal lymphatic vessels. Most splenic lymphangiomas are diagnosed in children. Ultrasound typically reveals splenic cysts of varying sizes, from a few millimeters to several centimeters in diameter. These cystic lesions appear as well-defined, anechoic or hypoechoic masses with occasional internal septa and intraluminal echogenic deposits. Splenic lymphangiomas are usually microcystic or solid and may exhibit central scarring. They are classified into three types: capillary, cavernous, and cystic [15]. A large, subcapsular, solitary cystic lesion incidentally detected in children, accompanied by internal septa and tiny wall nodules, suggests a diagnosis of lymphangioma [15]. Other organs may also be affected in the context of lymphangiomatosis.

**Figure 6:**
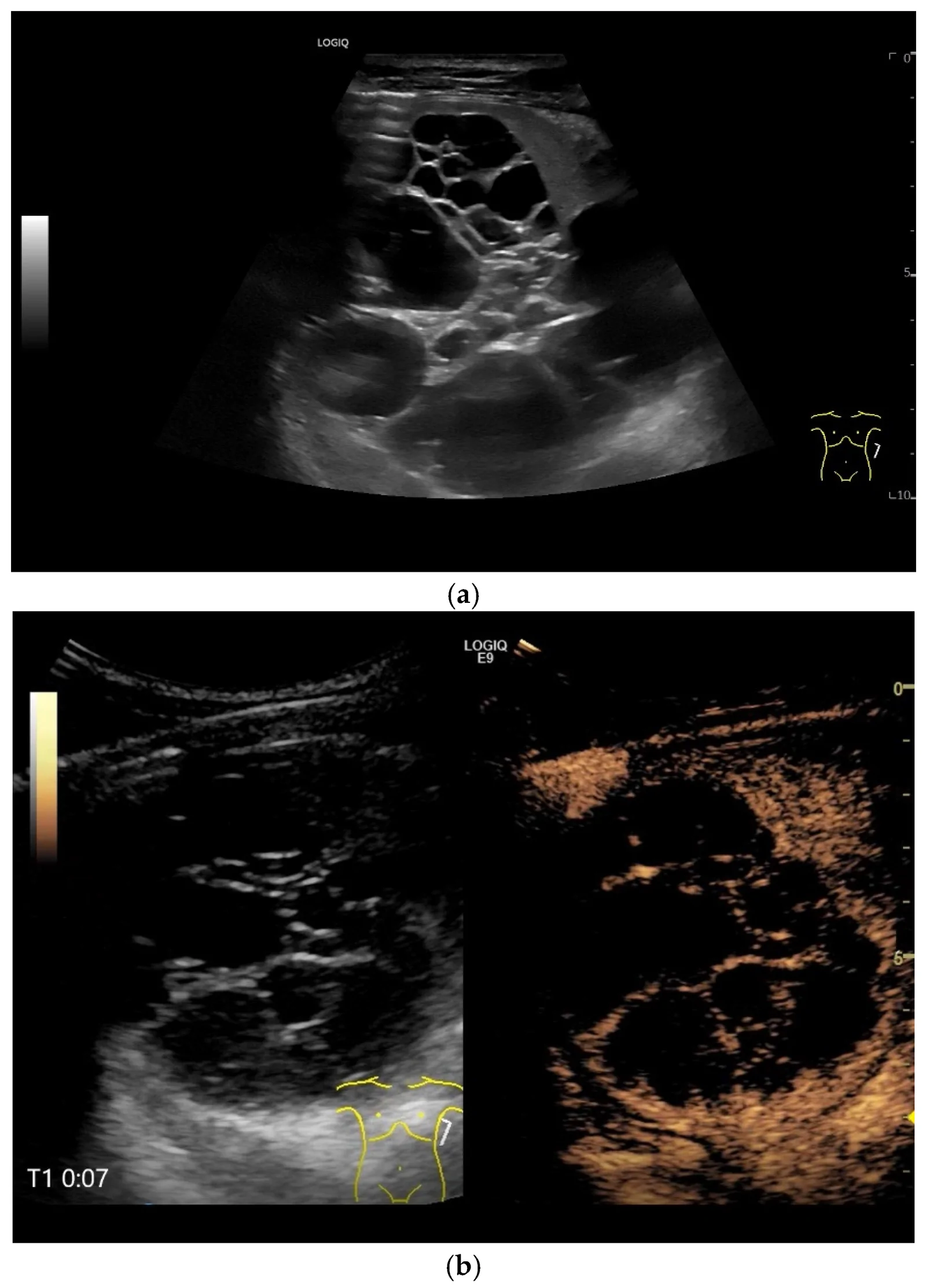
Lymphangioma. The patient presented with non-specific upper abdominal pain, no history of trauma, and an 80 mm thin-walled cystic splenic lesion with multiple internal septa (**a**). These septa were contrast-enhancing (**b**) on CEUS without washout. Echinococcus serology was negative. The diagnosis was a lobulated cystic lesion, most likely a lymphangioma, or alternatively a congenital cyst. Annual follow-up examinations over four years showed no changes in the findings.

Yu et al. described a hyperechoic lymphangioma with hyperenhancement in the arterial phase, rapid wash-in, and slow washout on CEUS [29]. An example of a chambered cystic lesion, which most closely resembles a lymphangioma, is shown in Figure 6.

### 4.5. Littoral Cell Angioma (LCA)

Littoral cell angioma (LCA) is a rare benign tumor of the spleen with malignant potential that originates from the (littoral) cells that line the sinus channels of the red pulp. A littoral cell angioma can only develop in the spleen, as littoral cells are found exclusively in the red pulp of the spleen. It was first described by Falk et al. [39]. In general, littoral cell tumors are classified into several categories, which include not only littoral cell angiomas but also littoral cell hemangioendothelioma (LCHE) and littoral cell angiosarcoma (LCAS) [40]. The cause of these lesions has not yet been fully established. A comorbidity rate of 13.8% with other tumors has been reported [40]. A relationship with other tumors or autoimmune diseases has been suggested, but there is not sufficient evidence to support this [40,41,42,43].

Wang et al. [40] analyzed 189 observational studies conducted up to 2021, involving 435 patients. These were primarily case reports. The diagnosis could generally only be made through histological examination. Only 31% of LCAs were solitary; multiple lesions were more common (ratio 0.31). Splenomegaly was observed in 69.7% of cases [40]. The study by Wang et al. [40] summarized the US characteristics of 92 patients with LCA. The LCA lesions appeared hyperechoic, hypoechoic, isoechoic, and heterogeneous in 43.5%, 35.9%, 9.8%, and 10.9% of patients, respectively. CDI results were available for 25 LCA patients. The lesions were described as hypovascular in 76% of cases and in 24% as hypervascular. CEUS was performed in eight patients. Arterial-phase contrast enhancement was observed in all eight patients. No description of the type of arterial contrast enhancement was provided. Of the 7 patients with a description of the parenchymal phase, 6 patients exhibited enhanced features on CEUS without further information on the specific enhancement pattern [40]. Görg et al. reported on two cases that appear on B-mode ultrasound as splenomegaly and multiple hyperechoic lesions. In their review, 6 out of 7 LCA cases were hyperechoic on ultrasound [44].

Cui et al. [41] reported a case of splenomegaly with multiple hypoechoic splenic lesions measuring up to 30 mm. No vessels were visible on the CDI. On CEUS, the lesions showed hyperenhancement in the arterial phase and hypoenhancement in the parenchymal phase. In the figure from the publication, there was mild hypoenhancement after 3 min [41]. Lerchbaumer et al. [45] reported on multiple isoechoic splenic lesions that could not be delineated on B-mode ultrasound and became visible only on CEUS. The lesions were hypoenhanced during the arterial and parenchymal phases of CEUS [45] [Table 4].

### 4.6. Sclerosing Angiomatoid Nodular Transformation (SANT)

**Figure 7:**
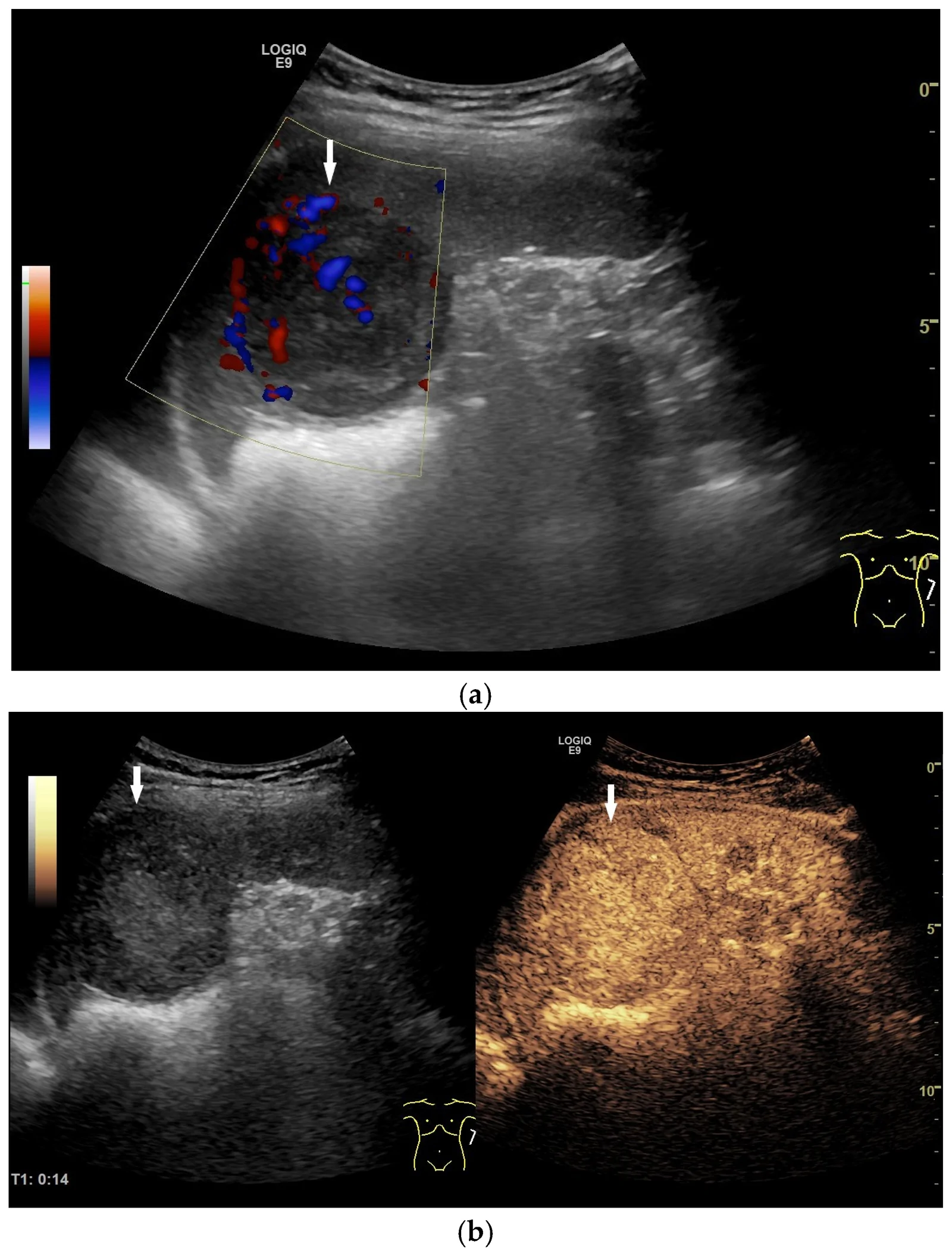

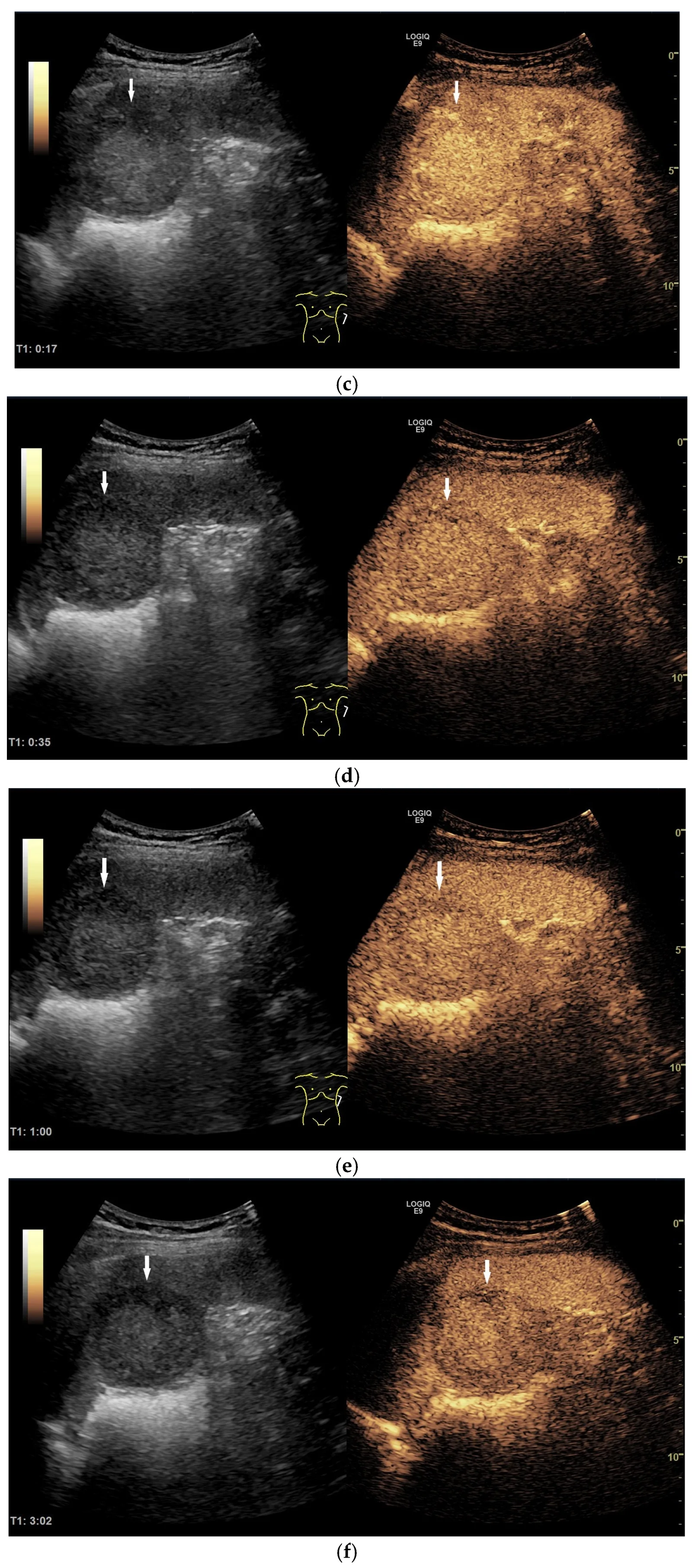
SANT/inflammatory pseudotumor (IPT). The patient presented with non-specific upper abdominal pain. An incidental finding was a 50 mm, slightly hypoechoic splenic lesion with numerous vascular reflections on CDI (indicated below with arrows) (**a**). On CEUS, the lesion initially showed mild inhomogeneous hyperenhancement in the arterial phase (**b**), followed by homogeneous hyperenhancement (**c**). In the beginning of the parenchymal phase, a washout with slight hypoenhancement was observed (**d**). At 1 min (**e**) and 3 min (**f**), the lesion showed slight hypoenhancement. MRI did not provide a definitive diagnosis. EUS-FNA was performed using a 22-gauge aspiration needle. The mini histology was inconclusive, and splenectomy was performed. Histological examination initially suggested SANT, but reference laboratory analysis indicated a more likely diagnosis of inflammatory pseudotumor.

Sclerosing angiomatoid nodular transformation (SANT) of the spleen is an extremely rare benign splenic lesion. The term “sclerosing angiomatoid nodular transformation” (SANT) was first coined by Martel et al. [46]. The authors described the histopathological appearance of this morphologically distinctive vascular tumor based on 25 cases. The solitary lesions were well defined and measured 3–17 cm in size. The tumors had a multinodular appearance, consisting of confluent nodules with an angiomatoid character and embedded in a dense fibrous stroma. Martel et al. hypothesized that these tumors were distinct, non-neoplastic vascular lesions of the spleen [46]. However, the exact etiology of SANT remains unclear; it is uncertain whether it is a de novo lesion or the final common pathway of other benign splenic conditions. It is hypothesized that SANT is a response to stromal proliferation and that the internodular zones resemble an inflammatory pseudotumor [46,47] [Figure 7]. Furthermore, an association with IgG4-related diseases [48,49] or with Epstein–Barr infection [50] is suspected. SANT has also been considered a variation of an inflammatory pseudotumor (IPT). SANT is characterized by fewer inflammatory cells and myofibroblasts than IPT [51]. A literature review of all 167 cases published in English up to 2018 and an analysis of 71 of these cases revealed that most patients were middle-aged women, and 15.5% of patients had a history of cancer. SANTs showed a tendency to grow during follow-up [52]. The growth rate of SANT is estimated at 0.75 mm per month, with a range of −0.08 to 1.77 mm per month [52]. On ultrasound, SANTs are described as a heterogeneous, hypoechoic or isoechoic mass with bright linear echoes corresponding to fibrous components [53,54,55]. CDI revealed small vessels in both the peripheral and central regions [51,55]. Gutzeit et al. reported a case of SANT with a vascular hypoechoic halo sign on B-mode ultrasound and a spoke wheel pattern on CEUS [53]. In contrast-enhanced CT and MRI studies, SANT of the spleen showed peripheral enhancing radiating lines [55]. Table 5 summarizes the enhancement patterns of SANT from case reports. SANT may demonstrate washout and parenchymal hypoenhancement and can therefore mimic malignant splenic lesions.

Further case descriptions of rare benign splenic tumors are listed in Table 6.

### 4.7. Sarcoidosis

Sarcoidosis is a systemic granulomatous disease characterized by non-necrotizing epithelioid cell granulomas. Sarcoidosis is not a disease that affects only the lungs and lymph nodes; it can also affect all other tissues, including the liver and the spleen [57]. The incidence of splenic involvement in sarcoidosis was reported to be 6.7% in a study of 736 patients [58]. Splenic involvement may be associated with splenomegaly. Sarcoidosis lesions are usually multiple and small.

Tana et al. [59] reported a case report involving splenomegaly, multiple hypoechoic lesions on B-mode ultrasound, and the absence of vascular signals on CDI. On CEUS, enhancement was slight and more peripherally located. Upon reviewing the figures, the lesions were predominantly hypoenhanced in the arterial phase, except for slight hyperenhancement in the peripheral areas. In the parenchymal phase, there was a rapid and marked washout with marked hypoenhancement [59].

Picardi et al. [4] described 20 patients with histologically confirmed sarcoidosis of the spleen, presenting with hypoenhancement in both the arterial and parenchymal phases.

Early hypoenhancement and progressive washout with marked hypoenhancement are criteria for malignant splenic lesions. The enhancement seen in sarcoidosis cannot be distinguished from that of malignant splenic lesions. Histological confirmation may be necessary when the diagnosis remains uncertain, particularly in patients without previously established sarcoidosis or when malignancy cannot be excluded. In patients with known systemic sarcoidosis and compatible clinical and imaging findings, additional tissue sampling may not always be required. This applies in particular to patients with no prior history of sarcoidosis.

**Figure 8:**
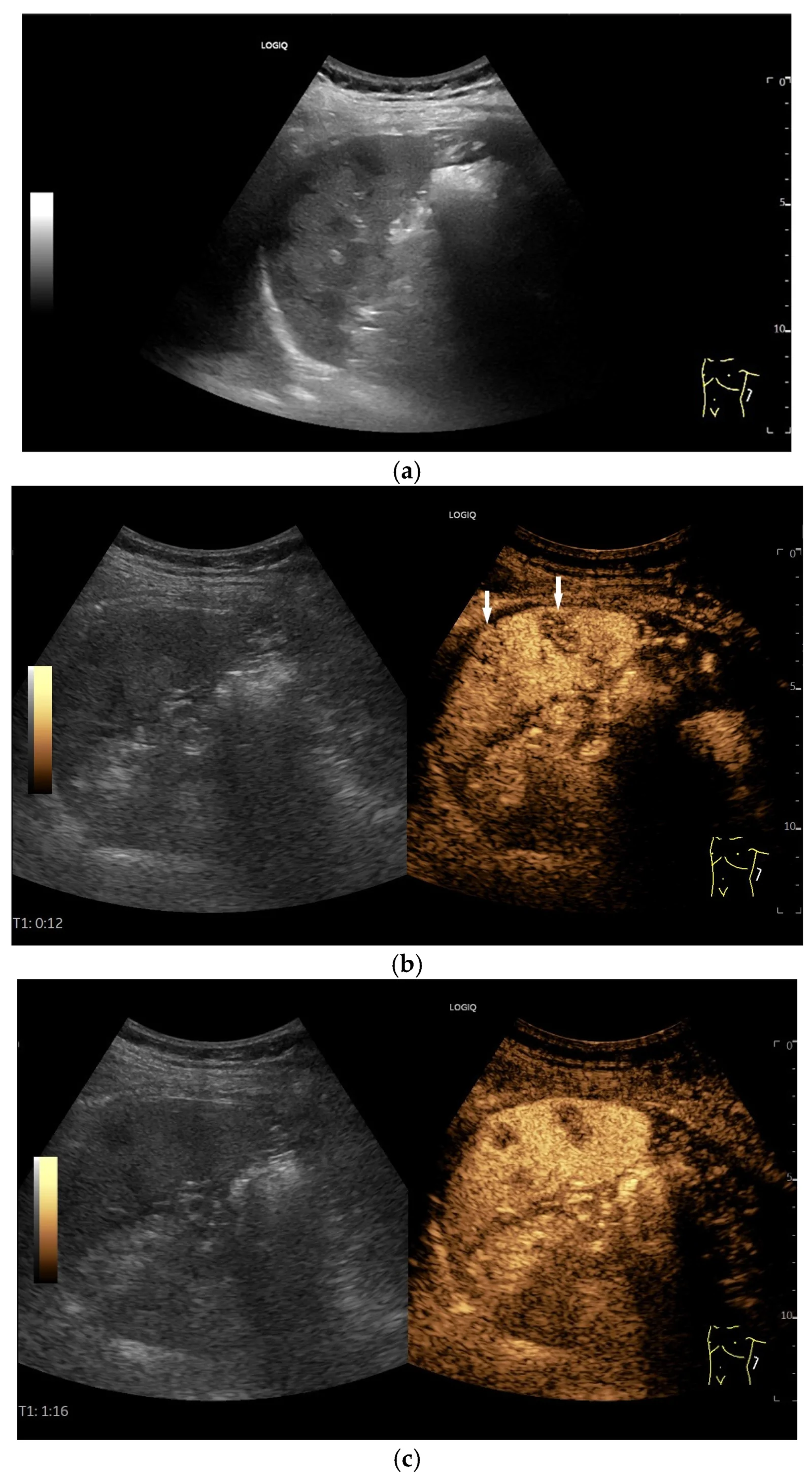

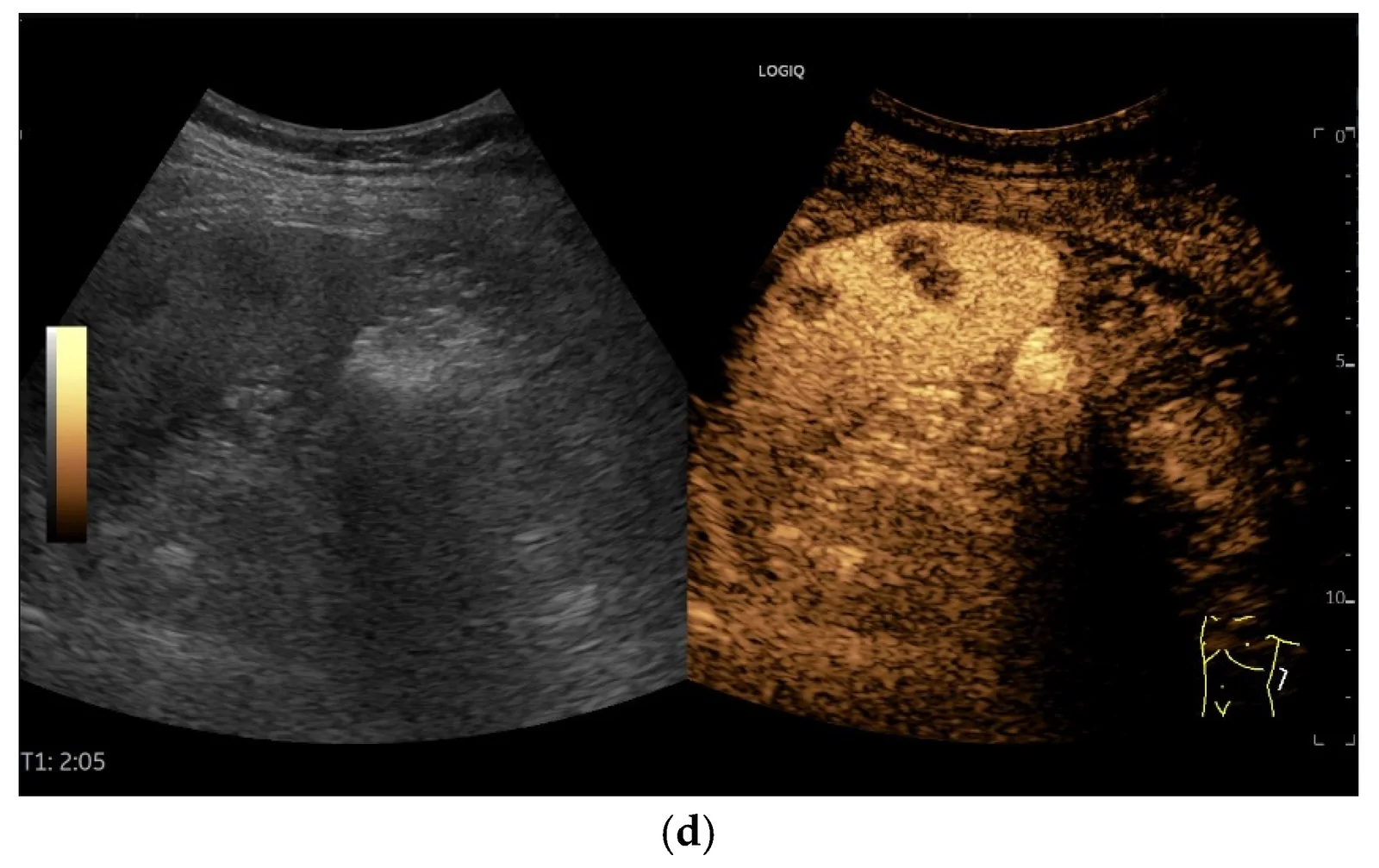
Sarcoidosis. During the staging of a newly diagnosed breast cancer, multiple mediastinal lymph nodes and hypoechoic splenic lesions were detected. Sarcoidosis was suspected, but tumor-related lymph nodes and splenic metastases could not be ruled out. The spleen was of normal size and exhibited multiple hypoechoic focal lesions (**a**). In the arterial phase (arrows) (**b**) and the parenchymal phase (**c**,**d**), hypoenhancement of the lesions with marked washout was observed. Histological confirmation of sarcoidosis was achieved via EUS-guided fine-needle biopsy (EUS-FNB) of the mediastinal lymph nodes (flexible 19G needle) and the splenic lesions (22G biopsy needle, Franseen tip).

**Figure 9:**
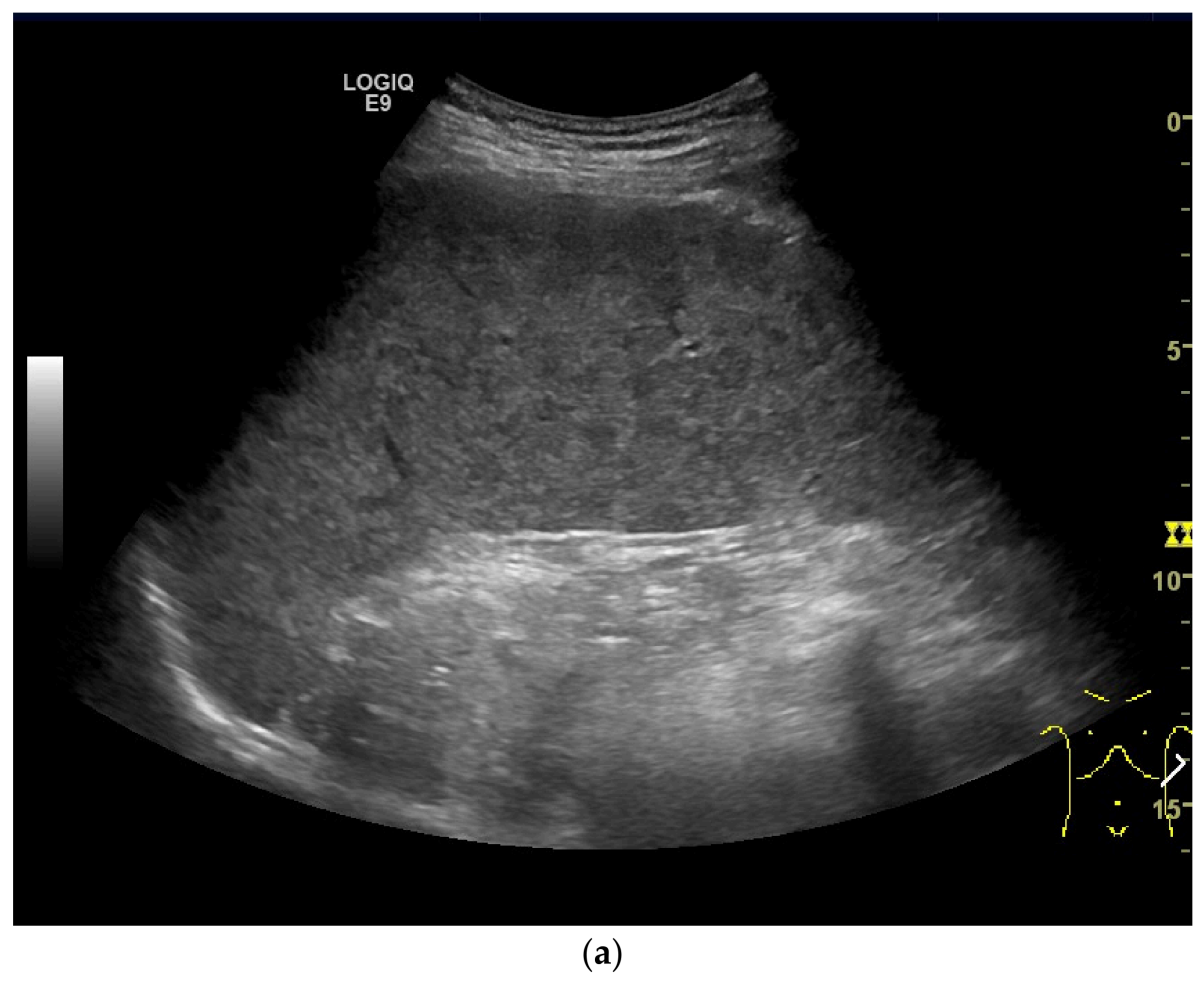

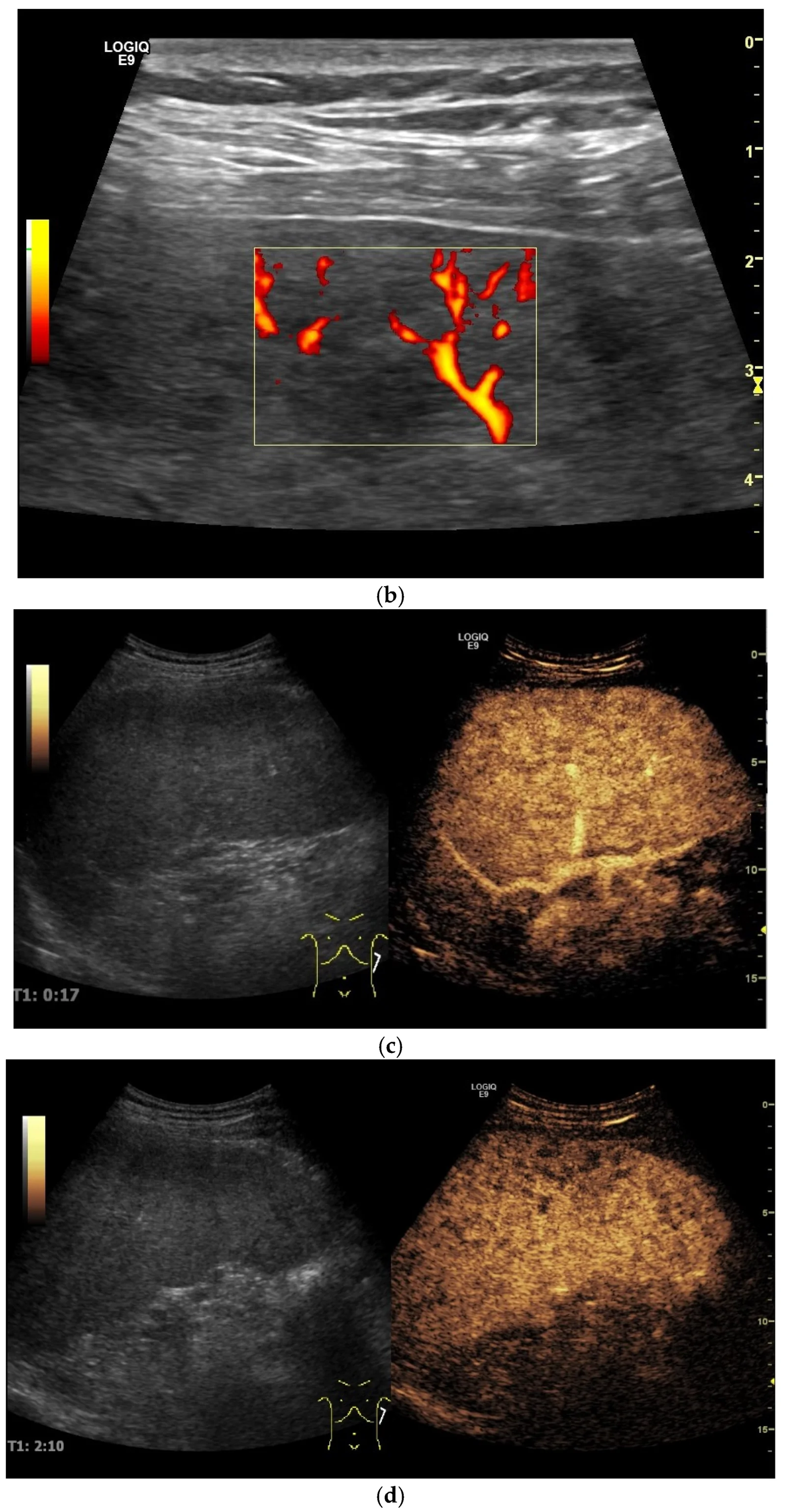
Sarcoidosis. Sarcoidosis with mediastinal lymphadenopathy and pulmonary and hepatic involvement was confirmed. Splenomegaly with disseminated finely patchy, hypoechoic, sub-centimeter focal lesions was also found (**a**). No vessels were detected in the lesions on CDI with power Doppler using a linear 9 MHz transducer (**b**). On CEUS, the lesions were hypoenhanced in the arterial phase (**c**) and showed progressive washout with marked hypoenhancement in the parenchymal phase (**d**). The findings were interpreted as splenic involvement of the known sarcoidosis.

Table 7 summarizes enhancement patterns of splenic sarcoidosis manifestations. Figure 8 and Figure 9 show typical images.

## 5. Malignant Splenic Lesions

### 5.1. Lymphoma

Lymphomas include non-Hodgkin lymphomas and Hodgkin lymphomas. Primary splenic lymphomas are rare. They are usually secondary splenic infiltrations in patients with a known lymphoma.

In cases of malignant lymphoma, splenomegaly is often evident on B-mode ultrasound. Diffuse splenic infiltration is difficult to detect on B-mode ultrasound. The B-mode ultrasound patterns described include diffuse infiltration; micronodular, large-nodular, and bulky disease; and perivascular involvement [63]. To better detect diffuse splenic infiltration and micronodular changes, it is recommended to evaluate the parenchyma using high-frequency linear transducers.

A typical feature in CEUS is hypo- or isoenhancement during the arterial phase, followed by washout and marked hypoenhancement during the parenchymal phase. Stang et al. [26] described 67 lymphoma patients who predominantly showed hypoenhancement in the arterial phase (59/67) and a few showed isoenhancement (8/67). The arterial enhancement was mostly heterogeneous, rarely homogeneous. In the parenchymal phase, all patients showed progressive and, in most cases, dotted hypoenhancement. Many patients exhibited atypical vessels within the lymphoma lesions [26].

In a study by Picardi et al. [4] of 194 lymphoma nodules in the spleen of 77 patients with non-Hodgkin lymphoma (NHL) and Hodgkin-lymphoma (HL), the typical finding in all patients (100%) was isoenhancement in the arterial phase with early progressive washout and hypoenhancement in the parenchymal phase. Washout started early, before 60 s post injection, at a median time of 43 s (20–60 s). The marked washout began at a median of 72 s (55–120 s). The marked washout occurred earlier in aggressive non-Hodgkin lymphoma (aNHL) than in classical Hodgkin lymphoma (cHL) and indolent non-Hodgkin lymphoma (iNHL). The range was 55–90 s (median, 74 s), 92–120 s (median, 100 s), and 101–120 s (median, 114.5 s). The average time to marked washout in the parenchymal phase was significantly shorter for aNHL compared with cHL (*p* < 0.001) and significantly shorter for cHL compared with iNHL (*p* < 0.001) [4]. The sensitivity, specificity, positive predictive value, negative predictive value, and overall diagnostic accuracy of CEUS in distinguishing splenic lesions as manifestations of lymphoma in at-risk patients with NHL or HD were 95%, 100%, 100%, 85%, and 96%, respectively. About one-quarter of the total lesions detected were other benign lesions [4].

The parenchymal phase of CEUS was of particular importance for the diagnosis and differentiation of lymphoma infiltrations in the spleen in patients at risk. The splenic lesions appeared in the parenchymal phase as clearly demarcated hypoenhanced lesions due to the rapid washout of the microbubbles compared to the otherwise homogeneously enhanced splenic parenchyma. This was attributed to the neoangiogenic vascular structure of lymphomatous lesions. It is hypothesized that angiogenesis played a different role in the various subtypes of splenic lymphomas. Aggressive NHL showed the most pronounced washout. This was attributed to the highest neoplastic microvascular density [4]. Washout timing may help stratify lymphoma aggressiveness.

**Figure 10:**
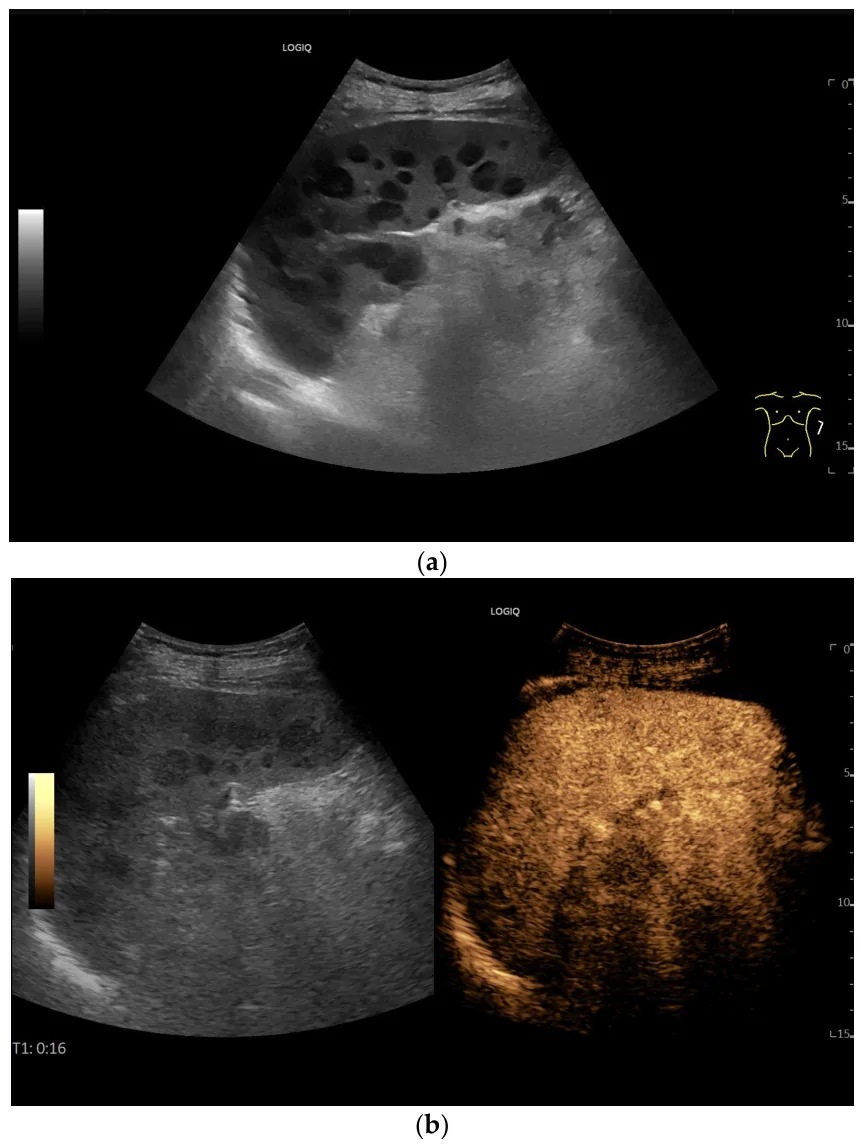

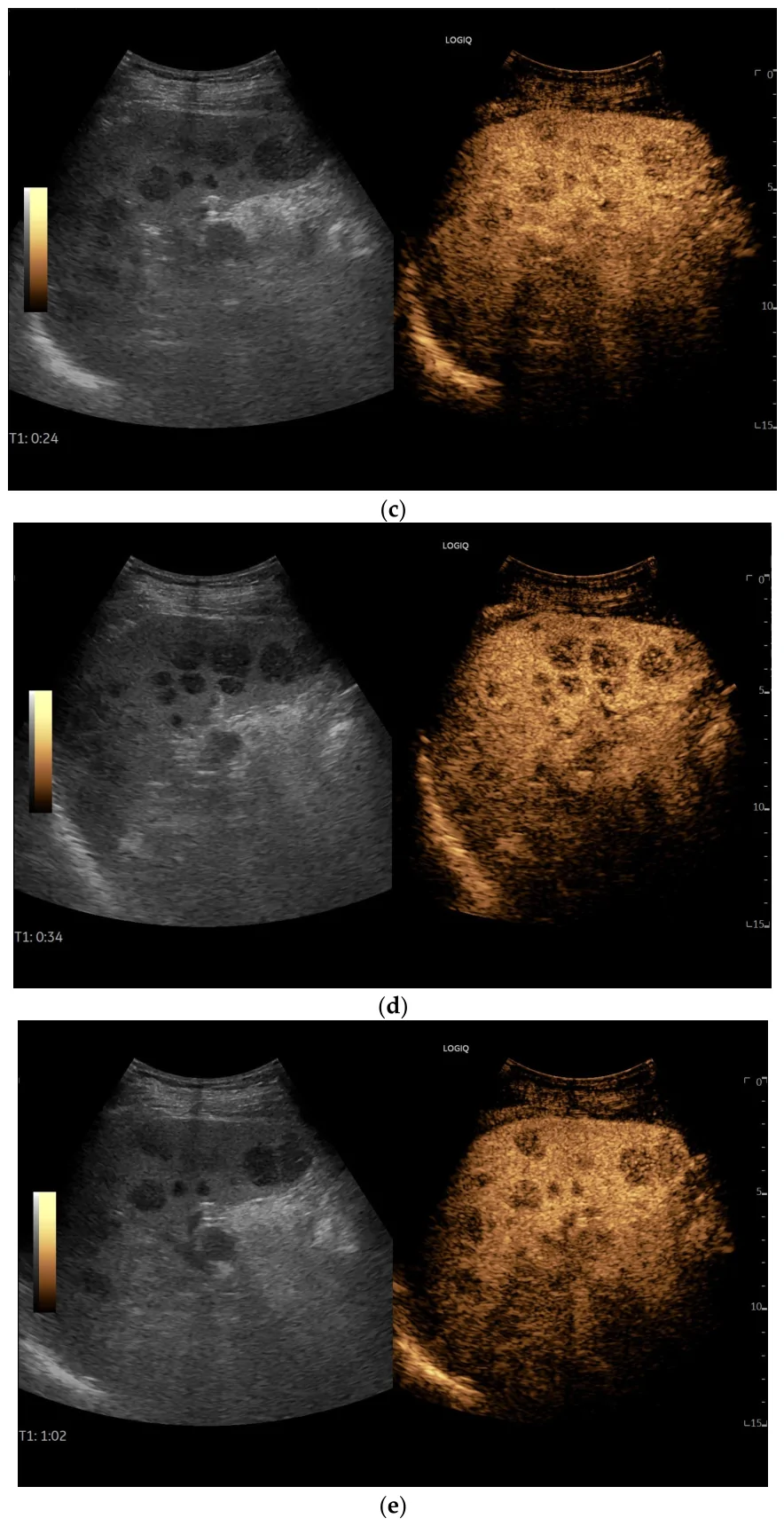

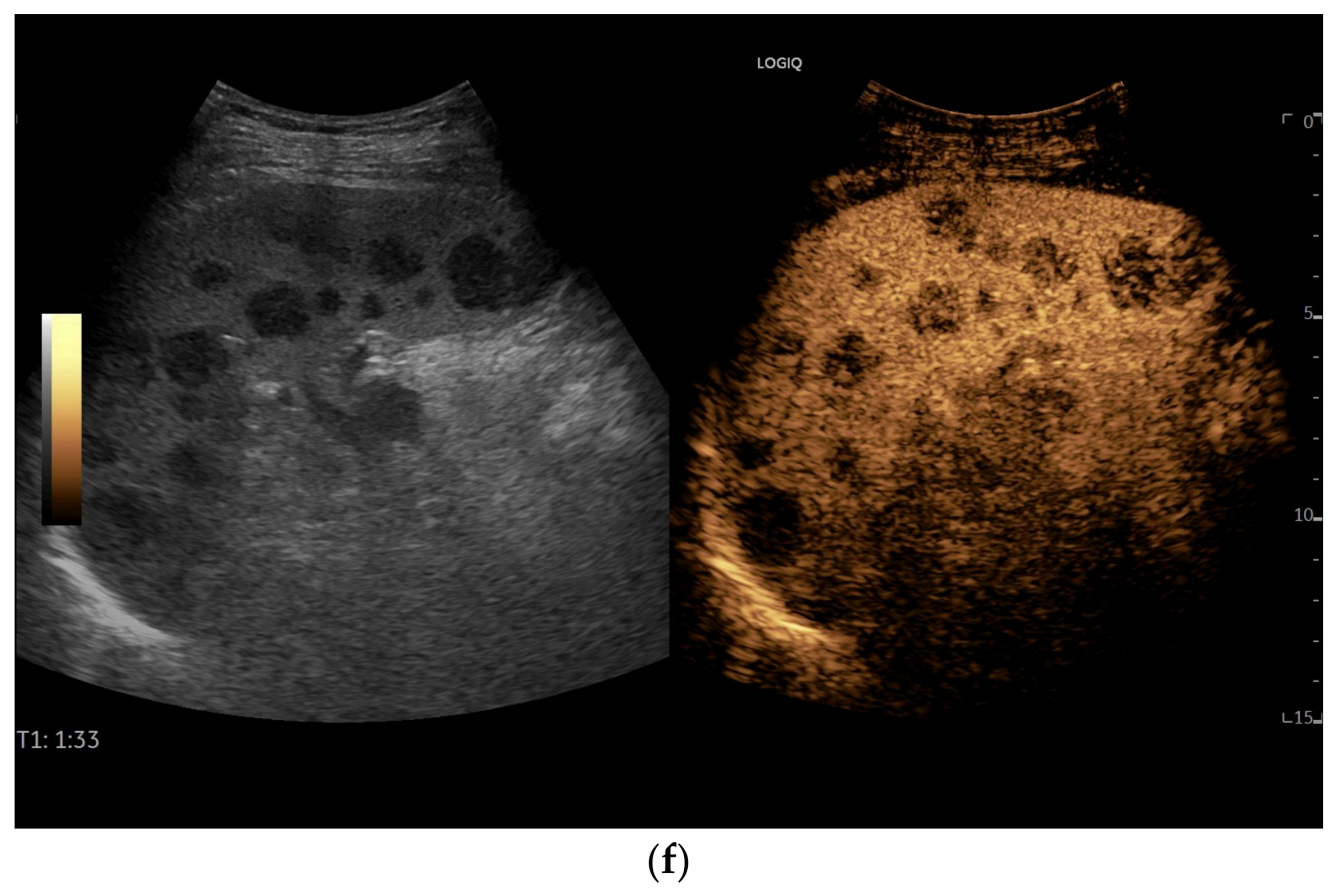
Aggressive diffuse large-cell NHL. Initial diagnosis was based on multilocular enlarged lymph nodes and mild splenomegaly (140 mm × 47 mm) with multiple hypoechoic lesions (**a**). On CEUS, iso- or mild hypoenhancement was observed in the arterial phase at 16 s (**b**), which increased at 24 s (**c**). A marked hypoenhancement developed as early as 34 s (**d**) and was also clearly detectable at 1.02 min and 1:33 min (**e**,**f**). Histological confirmation was obtained through ultrasound-guided sampling from a superficial lymph node using a 16 G needle.

**Figure 11:**
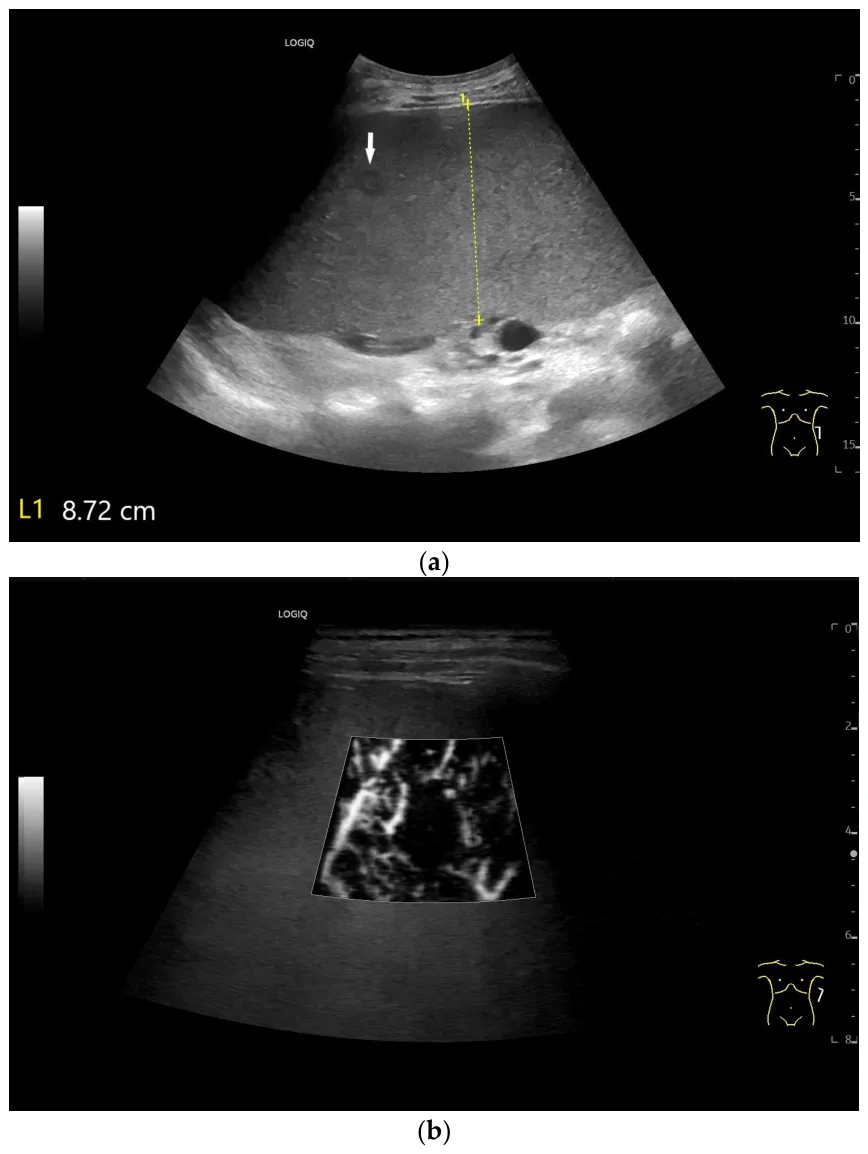

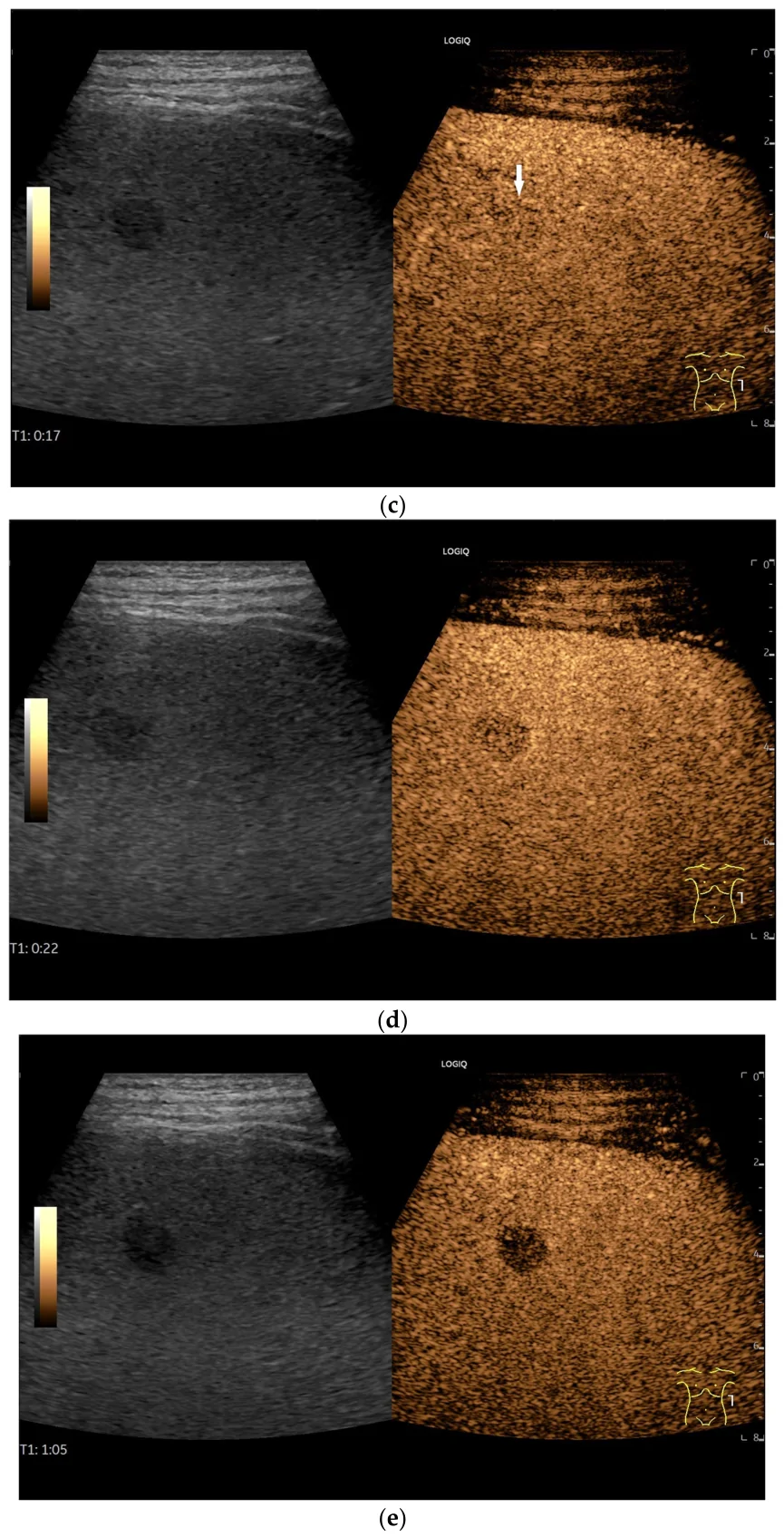

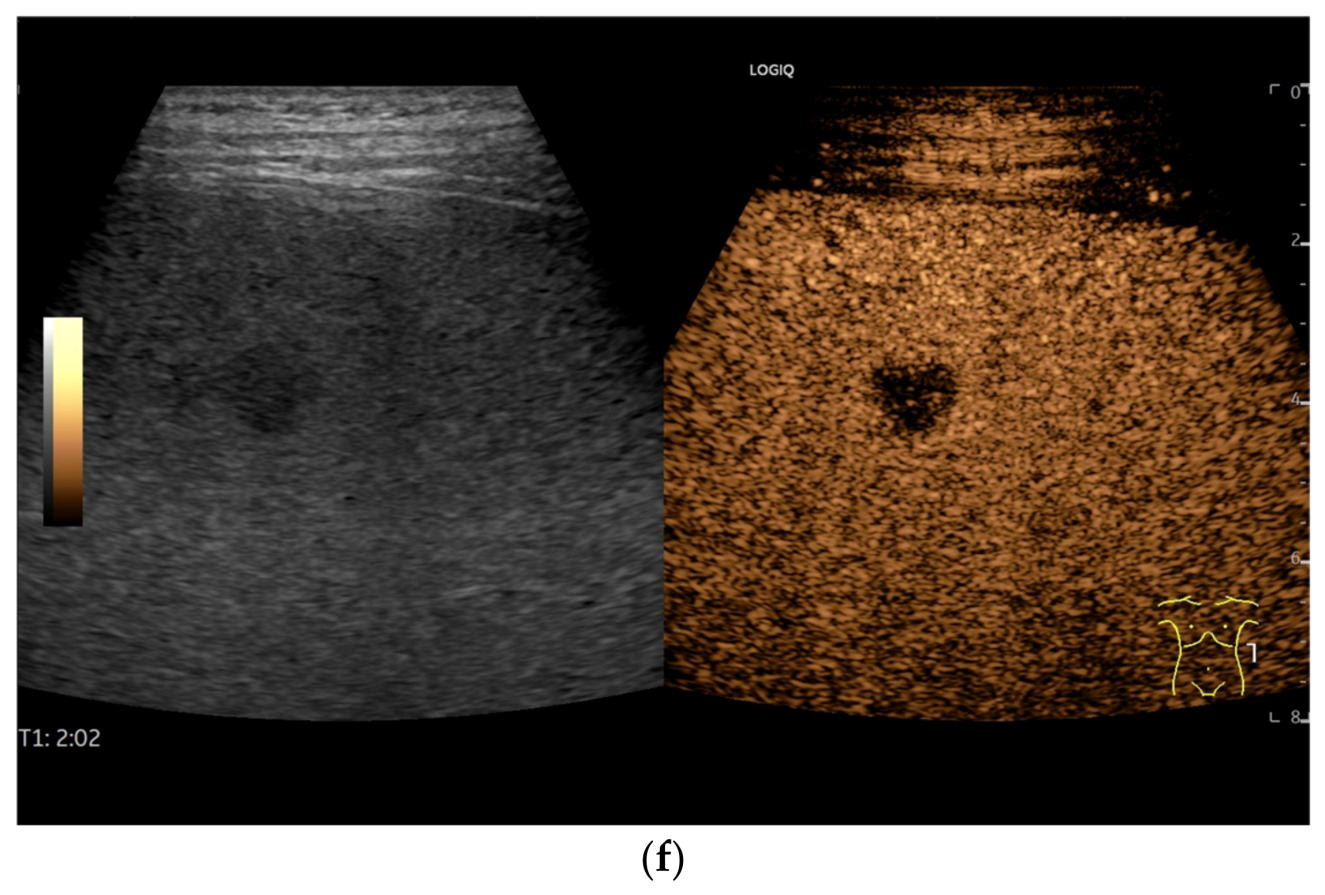
Previously indolent follicular NHL and splenomegaly with a hypoechoic splenic lesion (arrow). The markings indicate the measurement of the enlarged depth diameter of the spleen. (**a**). Microvascular imaging reveals no vessels within the lesion (**b**). In the arterial phase at 17 s, the lesion shows isoenhancement (arrow) (**c**); at 22 s, it shows mild hypoenhancement (**d**). Marked hypoenhancement becomes apparent only after 1.05 min (**e**) and continues to increase over time (**f**).

**Figure 12:**
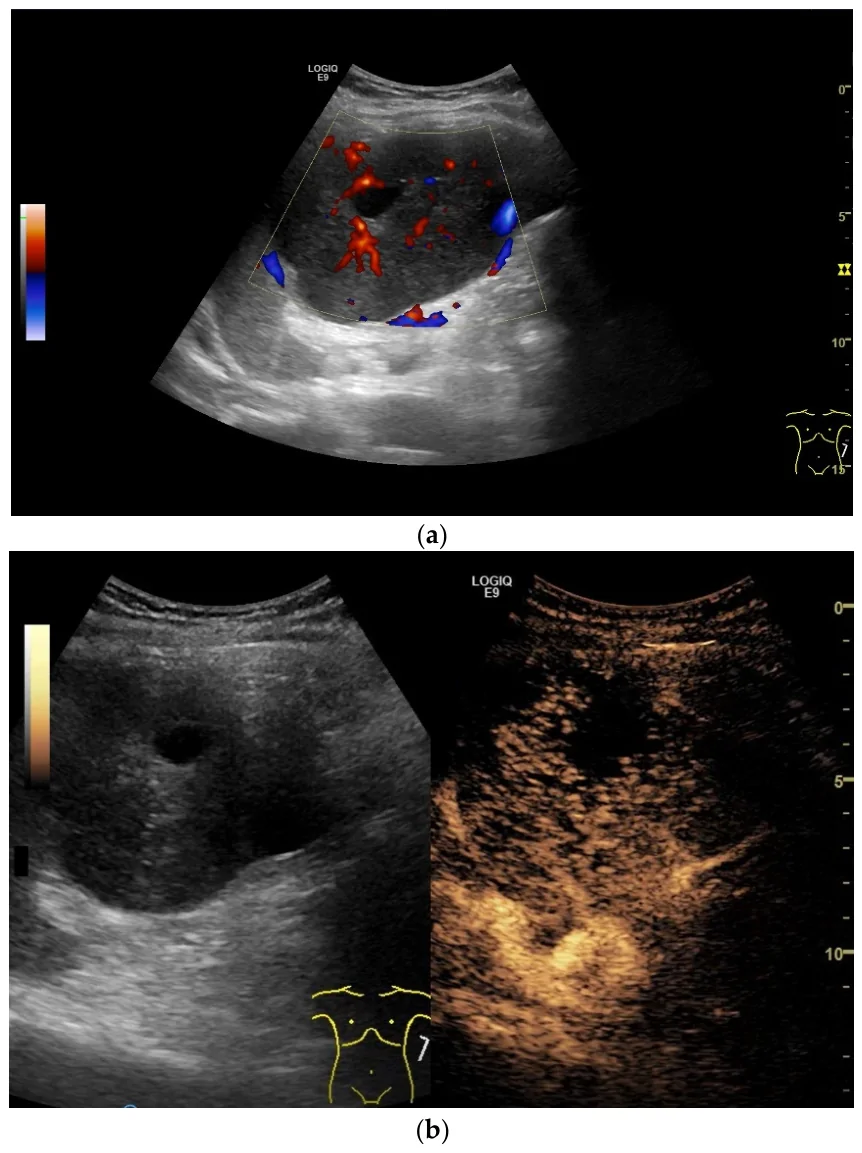

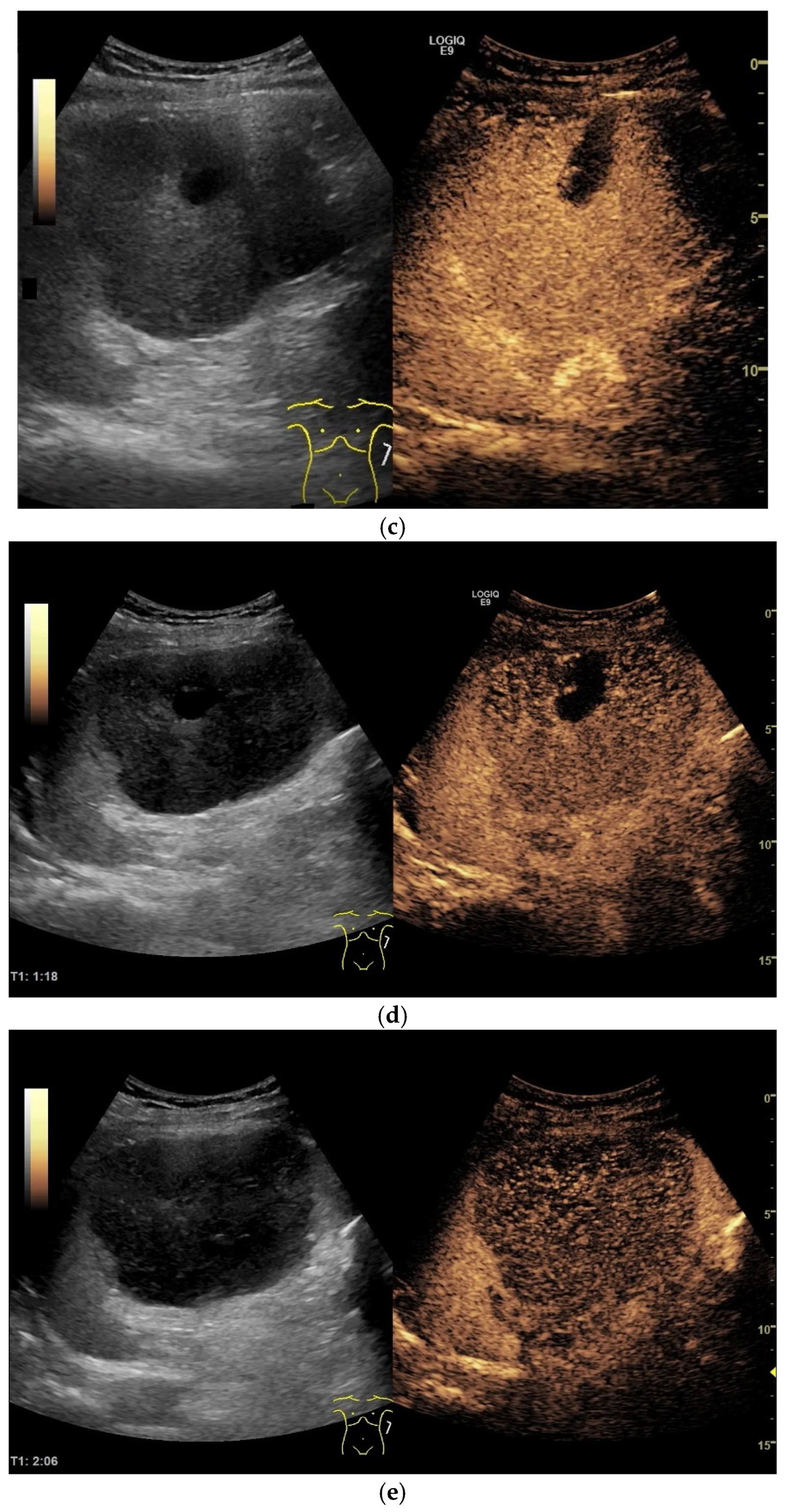
Primary diffuse large B-cell lymphoma. Initial diagnosis was made due to fatigue and non-specific abdominal symptoms. A smoothly demarcated, hypoechoic lesion with a central cystic component and vessels in solid parts can be observed (**a**). There was early arterial enhancement (**b**) and homogeneous isoenhancement in the arterial phase (**c**), followed by progressive washout in the parenchymal phase with the development of marked hypoenhancement (**d**,**e**). Histological confirmation was achieved through percutaneous US-guided sampling with an 18 G needle.

**Figure 13:**
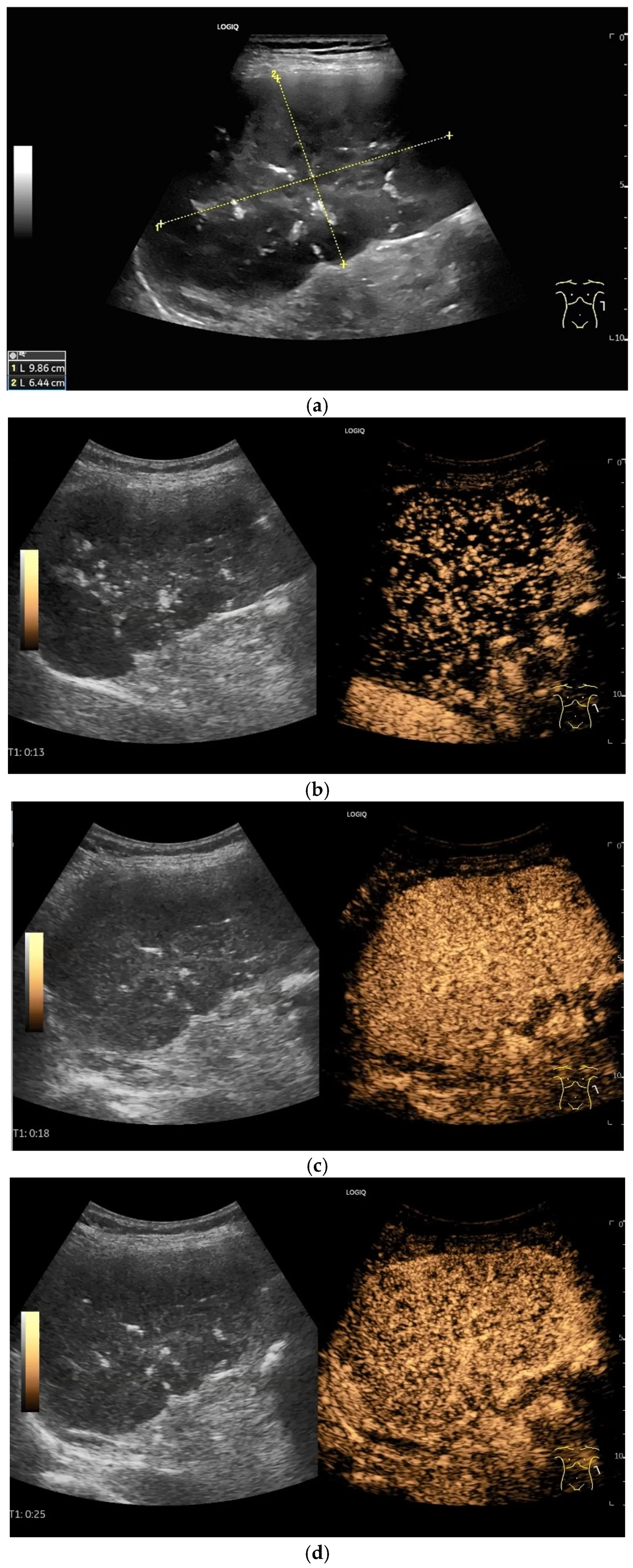

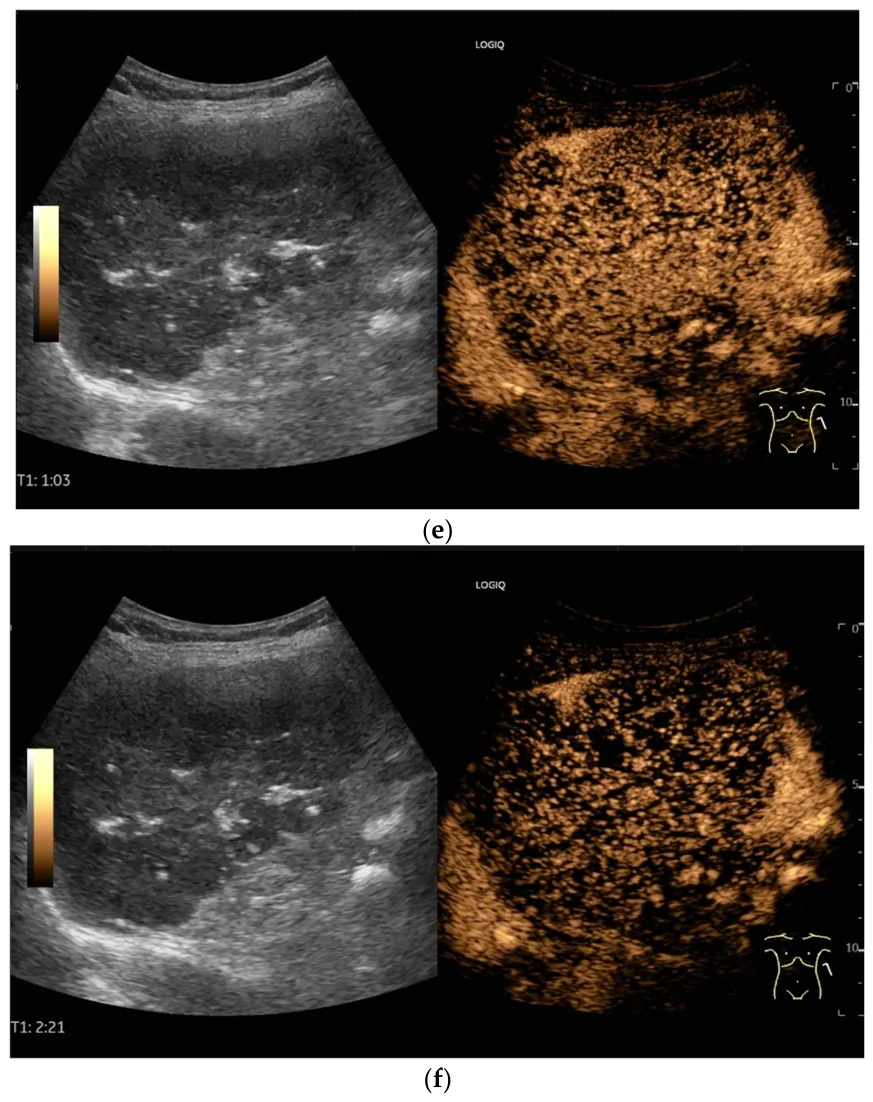
Diffuse large B-cell lymphoma. The patient presented with weight loss, fatigue, anemia, and non-specific abdominal symptoms. Splenomegaly was found with a 98 mm, poorly defined, hypoechoic lesion containing multiple highly echogenic internal structures (between the markers) (**a**). Enhancement in the early arterial phase on CEUS (**b**) and homogeneous isoenhancement in the arterial phase (**c**). At the end of the arterial phase at 25 s, a shift towards hypoenhancement began (**d**) and progressed to marked hypoenhancement in the parenchymal phase (**e**,**f**). Histological confirmation was obtained through US-guided sampling (18 G needle) of a pathological abdominal lymph node.

Zhang et al. [64] described splenic infiltration by diffuse large B-cell lymphoma (DLBCL) in a case report. The hypoechoic lesion was in the arterial phase, heterogeneous, and hypoenhanced. The lesion demonstrated a contrast enhancement pattern from the periphery to the center, with irregular hypoenhancement and marked washout and hypoenhancement [64]. Study data with characteristics of splenic lymphomas are summarized in Table 8 [Figure 10, Figure 11, Figure 12 and Figure 13].

### 5.2. Metastases

Splenic metastases are rare. Out of 168,000 ultrasound examinations, 59 splenic metastases (0.034%) were detected [71]. Some 95% of these patients already had metastases in other organ systems [71]. In further study with 50,000 abdominal ultrasounds including <0.6% focal splenic lesions, splenic metastasis was suspected in 32 patients (0.064%) [1].

Among 7246 individuals who died at a Swedish hospital (44.5% of postmortem examinations), 8214 primary malignant tumors were identified. Of these, 7165 were carcinomas. Splenic metastases were detected in 7.1% of the autopsies [72]. Microscopically distinct types of metastases have been described in the spleen—in the red and/or white pulp, in venous sinusoids, in the trabecular lymphatics, and in various combinations of infiltration [72]. In 1898 autopsies performed at the University of Marburg on patients with a solid malignant tumor, splenic metastases were diagnosed in 3.0% of cases [73].

On B-mode ultrasound, splenic metastases may appear hypoechoic, isoechoic, hyperechoic or with mixed echogenicity. Some 31% exhibited a hypoechoic rim [71].

In the study by von Herbay et al. [19], metastases (n = 6) exhibited isoenhancement, hyperenhancement, moderate hypoenhancement, and hypoenhancement/non-enhancement during the arterial phase. This was followed by early and marked washout. After 60 s, only one lesion showed moderate hypoenhancement, while in five patients, marked hypoenhancement or non-enhancement was observed. After 120–180 s, all metastases showed marked hypoenhancement/non-enhancement.

Neesse et al. [1] described exclusively the appearance of splenic metastases from a wide variety of primary tumors. In B-mode, the majority were hypoechoic (44%) or isoechoic (38%), but also hyperechoic (18%). In the arterial phase of CEUS, the majority were hypoenhanced (66%), a few were isoenhanced (6%), and some were also hyperenhanced (3%). A further 25% showed complex enhancement patterns with non-enhancing areas (necrosis) and/or rim enhancement in the arterial phase. In the parenchymal phase, 75% of all lesions showed hypoenhancement, and 25% complex enhancement patterns. Most metastases exhibited hypoenhancement in the arterial and parenchymal phases. Hyperenhancement in the arterial phase is rare in metastases but does occur and therefore does not rule out metastases [1]. The lesions were localized in the advanced primary tumor stage, and histological confirmation of splenic metastases was available in only one patient. No information was available on the primary tumor in the case of focal splenic lesion with hyperenhancement in the arterial phase.

**Figure 14:**
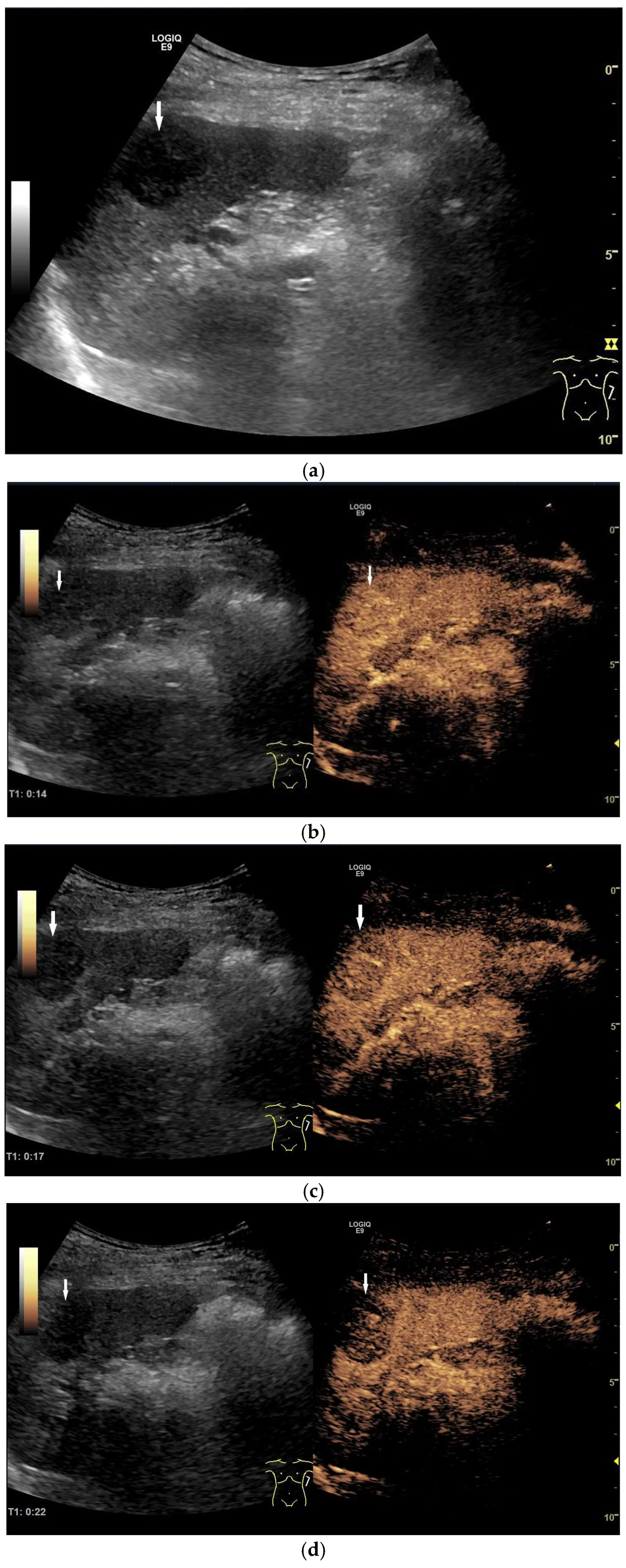

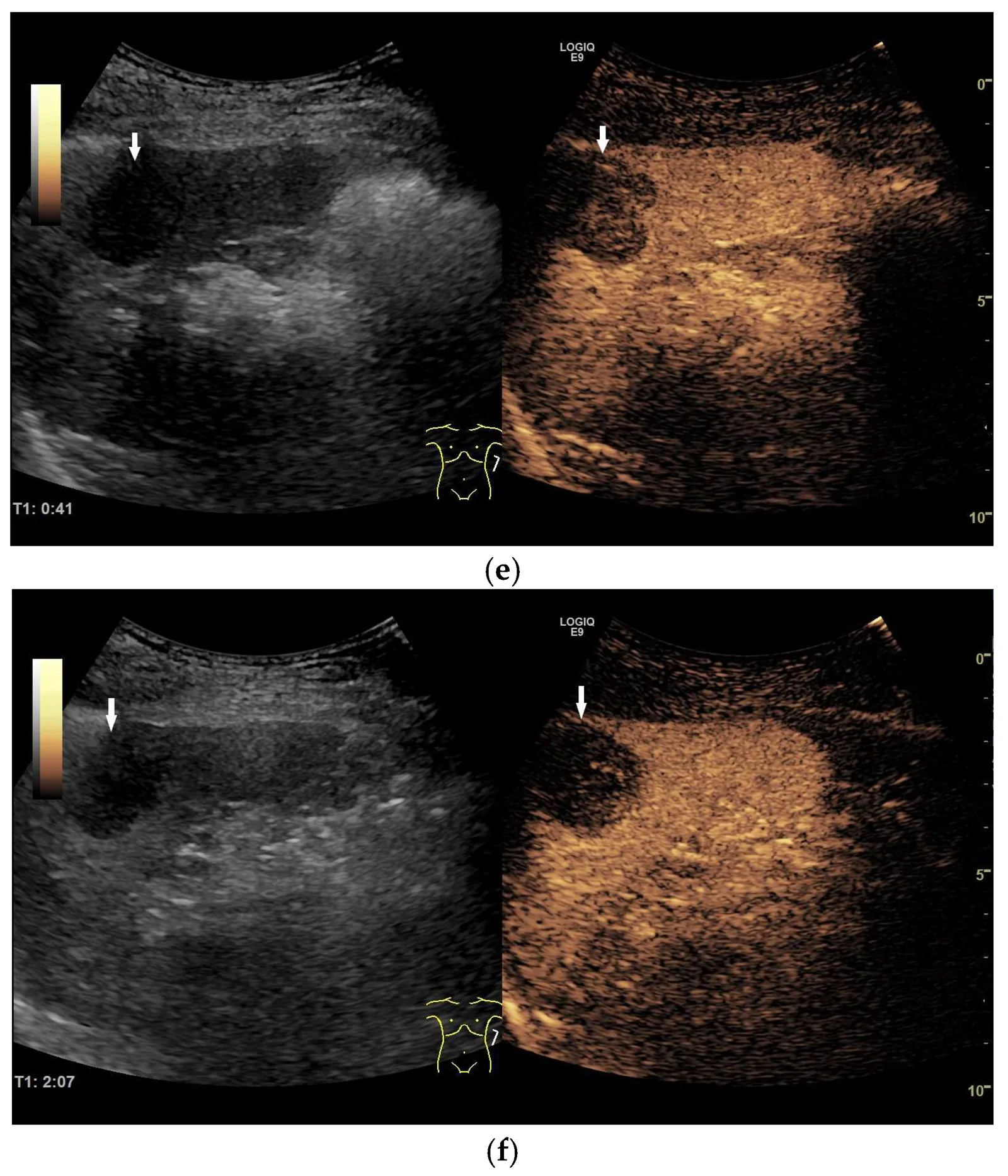
Metastasis of colon carcinoma. During staging at the initial diagnosis of a colon carcinoma, a hypoechoic splenic lesion was noted (as indicated below with arrows) (**a**). In CEUS, this lesion showed isoenhancement in the early arterial phase (arrow) (**b**). However, being already in the arterial phase, a marked washout began with the development of hypoenhancement (**c**,**d**), which continued progressively in the parenchymal phase (**e**,**f**). Splenectomy was performed concurrently with hemicolectomy, which histologically confirmed the presence of splenic metastasis.

The enhancement pattern was mostly heterogeneous, less frequently homogeneous, and (unlike liver metastases) only occasionally rim-like [26,29]. Study data with the characteristics of splenic metastases are summarized in Table 9 [Figure 14].

### 5.3. Hemangioendothelioma

Hemangioendothelioma is a vascular tumor with variable or unclear biological behavior. An epithelioid hemangioendothelioma is described as a hypoechoic lesion on B-mode ultrasound, with heterogeneous hypoenhancement on contrast-enhanced ultrasound (CEUS) during the arterial phase and marked washout and hypoenhancement during the parenchymal phase [29].

### 5.4. (Hem-)Angiosarcoma

Despite being rare, angiosarcoma is the most commonly occurring malignant primary vascular neoplasm of the spleen [15,75].

Yu et al. [29] described a hemangiosarcoma. This was a large hypoechoic lesion associated with splenomegaly. On contrast-enhanced ultrasound (CEUS), the lesion showed heterogeneous hyperenhancement alongside non-enhancing necrotic areas. The solid components with enhancement exhibited rapid wash-in and rapid washout pattern, followed by the development of marked hypoenhancement [29]. In two very rare cases of myxofibrosarcoma and Langerhans’ cell histiocytosis, hypoenhancement in the arterial and late phases has been described [34].

## 6. Differential Diagnosis of Benign and Malignant Splenic Lesions on CEUS

Unlike liver lesions, there are no sufficiently reliable and typical vascular patterns for focal splenic lesions. In contrast to national multicenter studies for liver lesions, only smaller single-center studies exist for splenic lesions. For rare splenic tumors, there are only isolated case reports on CEUS enhancement.

Yu et al. [29] described four major enhancement patterns for benign lesions: Rapid wash-in and slow washout pattern (48%); rapid wash-in and rapid washout (19%); isoenhancement and slow washout (10%); and isoenhancement and rapid washout (10%) [29]. However, rapid, marked washout is also characteristic of malignant lesions. While lymphomas predominantly show hypo or isoenhancement in the arterial phase, metastases may also exhibit hyperenhancement in the arterial phase.

Von Herbay et al. reported that homogeneous (in figures = isoenhancement), heterogeneous, or hypervascularized (= hyperenhancement) contrast enhancement in the early phase (1–20 s after injection), followed by rapid washout and clear demarcation 60 s after injection, was observed in 10 of 14 malignant lesions but in only 1 benign lesion (hemophagocytic syndrome; *p* < 0.001; sensitivity 0.71; specificity 0.95; positive predictive value 0.91; and negative predictive value 0.83). 

Completely non-enhancing lesions were observed only among benign conditions in the cited study, including infarctions, cysts, hematomas, and the non-perfused components of abscesses. Hyperenhancement in the parenchymal phase did not occur in any malignant lesions but exclusively in a benign hamartoma [19].

In a study by Bert et al. [76], a hyper/isoenhancement with central or peripheral contrast enhancement during the arterial phase was consistent with vascular tumors.

In a study by Stang et al. [26], benign lesions in the arterial phase showed non-enhancement in 31 of 68 lesions, isoenhancement in 26 of 68, and hypoenhancement in 11 of 68. The same figures were reported for the parenchymal phase. In the arterial phase, 8 out of 79 malignant lesions were isoenhanced and 71 out of 79 were hypoenhanced. All malignant lesions showed hypoenhancement in the parenchymal phase. Non-enhancement in both phases and isoenhancement during the parenchymal phase were characteristic of benign lesions [26].

In further study by Stang et al. [38] with 58/136 benign splenic lesions and 78/136 malignant splenic lesions, arterial phase hyperenhancement was predominantly observed in benign lesions, and arterial phase hypoenhancement was predominantly observed in malignant lesions. In the multivariate assessment, arterial hyper or isoenhancement were independent determinants of benign vascular neoplasia (odds ratio, 3.558; *p* < 0.0017). In the subgroup of iso or hypoechoic lesions, arterial hyperenhancement was conclusive for a benign vascular neoplasm (odds ratio, 21.333; *p* < 0.001) [38]. Arterial isoenhancement was described in both benign and malignant lesions. Hyperenhancement in the arterial phase with isoenhancement in the parenchymal phase and lesions with isoenhancement in both phases were exclusively associated with benignity. Lesions with hyperenhancement in the arterial phase and hypoenhancement in the parenchymal phase were predominantly benign, although malignant lesions were present. For lesions with isoenhancement in the arterial phase and hypoenhancement in the parenchymal phase, the proportion of malignant lesions increased significantly. Lesions with hypoenhancement in both the arterial and parenchymal phases were predominantly malignant, although there were exceptions of benign lesions in this group as well [38]. Although hyperechoic lesions tended to be benign (*p* = 0.0030) and hypoechoic lesions were more frequently malignant (*p* < 0.001), there were no hyperechoic lesions with arterial phase hyperenhancement in this study, neither among benign nor malignant lesions [38]. In a study by Götzberger et al. involving 38 splenic lesions, hyper/isoenhancement in the arterial phase was observed in 90% of the benign solid lesions but not in any of the malignant lesions. Rapid washout was seen in 100% of the malignant lesions but only in 7% of the benign lesions. Non-enhancement was a characteristic exclusively of benign lesions. A correct assessment of malignancy using contrast-enhanced ultrasound (CEUS) was possible in 89% of the lesions [77].

In a study by Lerchbaumer et al. [78], all malignant lesions (n = 5) demonstrated washout. This was the only significant difference (*p* < 0.001) compared to 30 benign lesions [78].

Yang et al. [79] analyzed splenic lesions from 123 patients. Exclusively benign lesions showed either non-enhancement (20.5%) or persistent hyperenhancement in the parenchymal phase (15.7%). Hyperenhancement in the arterial phase was more common among benign (38.6%) than malignant (2.5%) lesions. Most malignant lesions (97.5%) showed hypoenhancement (without phase assignment) compared to approximately one-quarter of benign lesions (25.3%). Only benign lesions were wedge-shaped [79].

In the study by Alhyari et al. [24], both benign and malignant focal splenic lesions showed arterial hyper, iso, or hypoenhancement, with no significant difference. While only benign lesions (66.7%) showed no washout or only mild washout in the parenchymal phase, all malignant lesions (100%) (as well as 33.3% of the benign lesions) exhibited marked washout [24].

**Figure 15:**
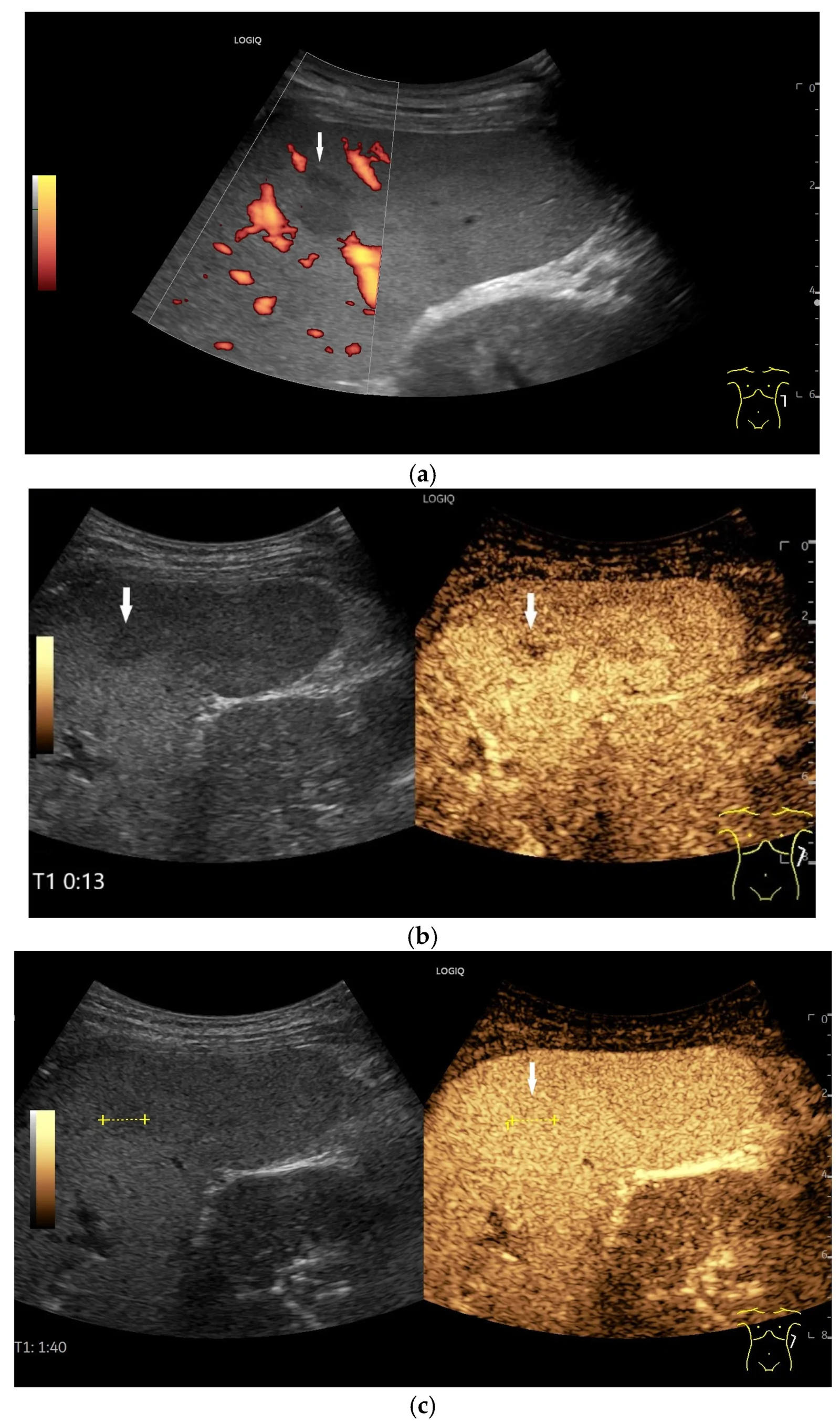

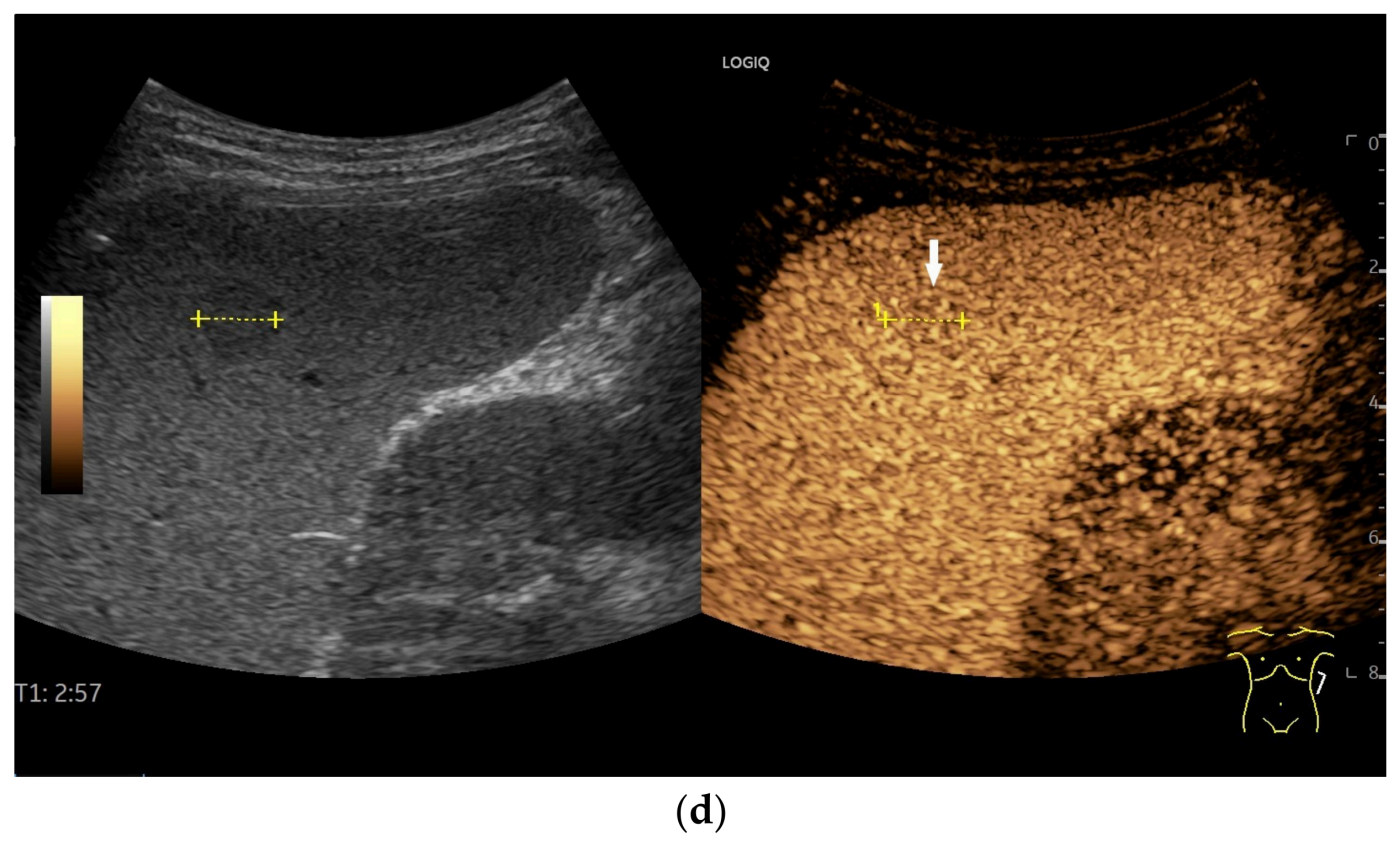
Benign splenic lesion on CEUS: Incidental finding of a 12 mm hypoechoic focal lesion (arrow) without detection of vessels on CDI with power Doppler in a patient with non-specific epigastric complaints (**a**). In the arterial phase on CEUS, hypoenhancement and delayed enhancement were observed (arrow) (**b**). In the parenchymal phase, isoenhancement and no washout were observed (between the markers and arrows) (**c**,**d**). The splenic lesion was classified as benign. Laboratory tests, an abdominal ultrasound, and a gastroscopy revealed no abnormalities.

**Figure 16:**
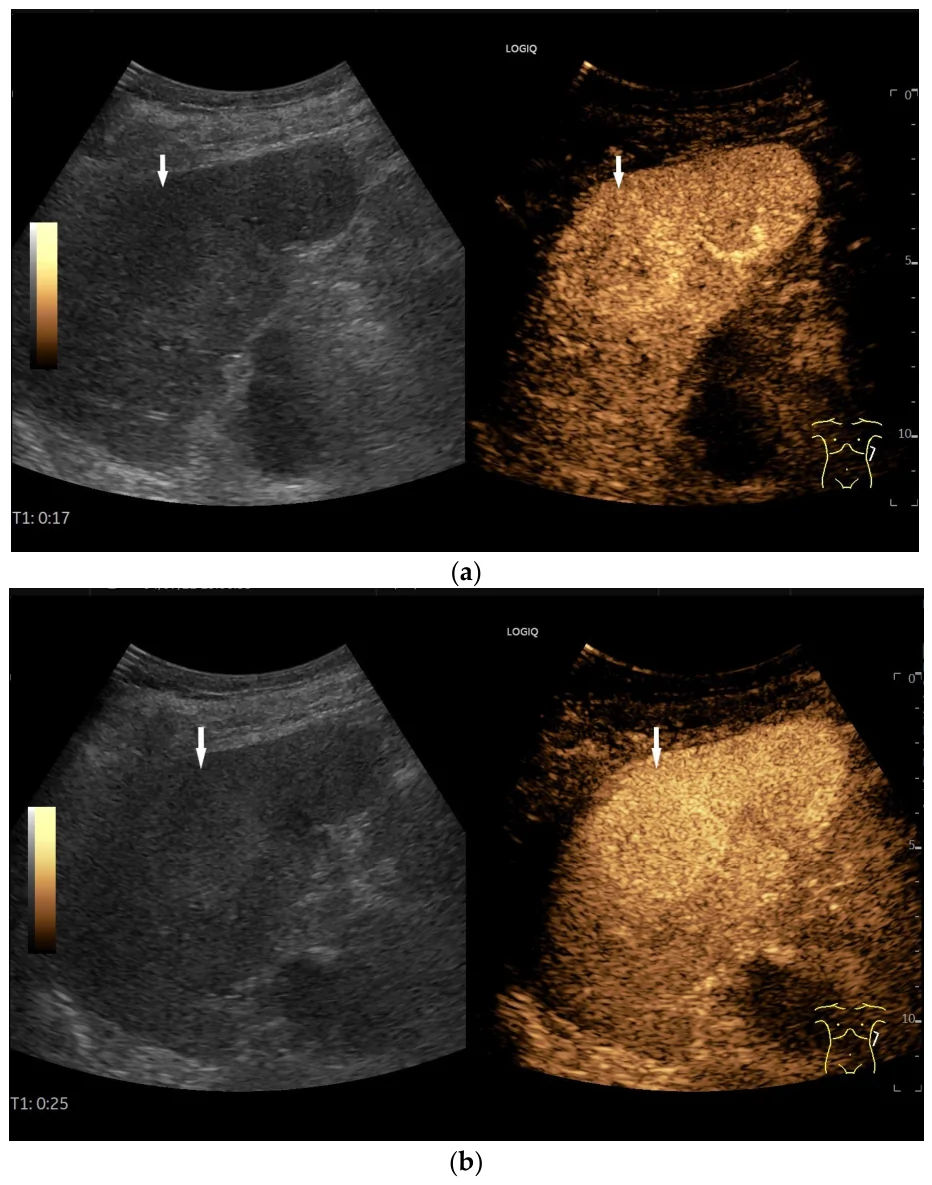

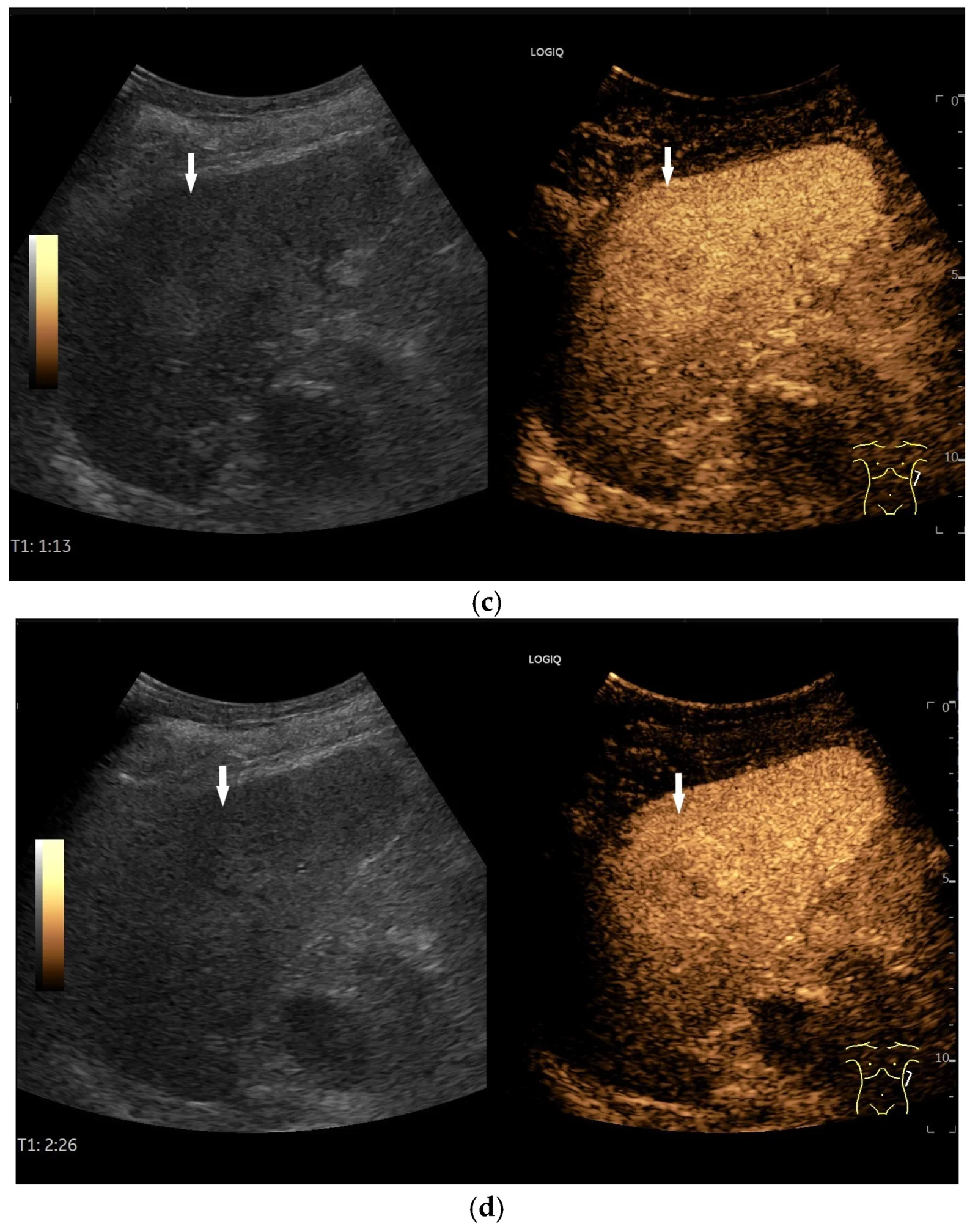
Benign lesion. A splenic lesion was incidentally discovered on chest CT during a pulmonary embolism. The lesion was slightly hypoechoic on B-mode ultrasound and showed peripheral hyperenhancement in the early arterial phase, while the central portions were isoenhanced/slightly hypoenhanced compared to the splenic parenchyma (**a**), as indicated below with arrows. By the end of the arterial phase, the lesion was homogeneously hyperenhanced (**b**). In the parenchymal phase, the central parts were initially isoenhanced, and the periphery remained hyperenhanced (**c**), followed by minimal washout with only faint hypoenhancement of the central parts of the lesion (**d**). No paraneoplastic process was diagnosed during the evaluation of pulmonary embolism, and the splenic lesion remained unchanged during follow-up.

In the study of Safai Zadeh et al. [2] of incidental splenic lesions (93.5% benign and 6.5% malignant), all lesions (both hyperechoic and hypoechoic in B-mode) that showed arterial phase hyperenhancement or absent enhancement were benign. Among lesions showing isoenhancement or hypoenhancement in the arterial phase, hyperechoic lesions were slightly more likely to be benign than hypoechoic lesions in B-mode (93.7% versus 83.8%) [2]. Table 10 summarizes various findings of different echogenicity in B-mode and enhancement in CEUS in benign and malignant lesions from different studies. Figure 15 and Figure 16 show cases in which benignity was determined using CEUS enhancement.

### Dynamic Contrast-Enhanced Ultrasound (DCE-US)

Unlike the subjective assessment of enhancement in CEUS, dynamic contrast-enhanced ultrasound (DCE-US) allows for a quantitative representation of blood flow by plotting a time–intensity curve (TIC) [80,81]. The curves from a region of interest (ROI) within the parenchymal lesion are compared with those from the normal adjacent parenchyma at the same penetration depth. The TICs show the phases of progressive increase in the enhancement of the contrast agent in the ROI (wash-in phase) and the subsequent phase of slow decrease in contrast signal intensity (washout phase). A distinction is made between time-related parameters and signal intensity-related parameters. Time-to-peak (TP), rise time (RT), mean transit time (MTT), peak intensity (PI) and area under the curve (AUC) are proposed as primary parameters. All others are derived from these parameters. To date, there are no quantified data available regarding a specific percentage decrease in enhancement over a given time period for benign and malignant splenic lesions. Thus, hyper- or hypoenhancement relative to the surrounding parenchyma can be objectively assessed in the TIC. An objective measurement of perfusion could serve as the basis for a standardized a future splenic CEUS reporting framework as a research objective.

Limitations include the patient’s respiratory movements, which can cause dislocation or displacement of the ROI, and, particularly in the case of the spleen, superimposition of lung artifacts.

## 7. US-Guided Sampling of Splenic Lesions

Splenic lesions that are unclear or suspected of being malignant in imaging with US, CEUS, or MRI can be histologically confirmed by US- or EUS-guided sampling [82,83,84,85], with several studies reporting a high diagnostic yield but a relevant rate of adverse events. Earlier studies using large-caliber needles (14 G) reported complication rates of up to 13% for splenic biopsies [86]. Currently, however, percutaneous US-guided sampling is performed using 18–25 G biopsy needles. In a study using US-guided sampling with 18 G needles of splenic nodular lesions suspected of hematologic disease in 44 patients, the diagnostic yield was 88.6% with a sensitivity of 96.6%, a specificity of 100%, and an accuracy of 86.4%. Some 50% of the splenic lesions were lymphomas [87]. In another study, specimens suitable for histological diagnosis were obtained using 18 G needles in 90.4% of 52 patients [88]. The largest cohort of percutaneous US- (n = 74) and CT-guided (n = 23) core needle biopsies of the spleen reported a diagnostic yield of 93.8% with a sensitivity of 90.7% and a specificity of 100% (accuracy of 94.5%) [89]. There were seven (7.2%) minor complications and a single (1.0%) major complication; the overall complication rate was 8.2% (n = 8). The diagnostic yield—defined as sufficient tissue to establish a diagnosis—of CNB was 93.8%; the sensitivity was 90.7%, the specificity was 100%, and the accuracy was 94.5%. A meta-analysis of 858 US-guided sampling procedures showed a sensitivity of 87.0% and a specificity of 96.4% [90]. In this meta-analysis, an overall complication rate of 4.2% (major: 2.2%) was reported for all splenic biopsies (5.8% for core needle biopsy with 3.2% major complications; 4.3% for fine needle aspiration or biopsy with 1.3% major complications) [90]. In a large German multicenter study of over 8000 patients undergoing US-guided abdominal interventions, 63 US-guided splenic samples were included, compared to 3400 US-guided liver samples. Two major complications (3.17%) occurred during splenic biopsy, compared to 0.56% after biopsy of focal liver lesions. The frequency of significant hemorrhages was 1.5% after splenic biopsies and 0.32% after biopsy of focal liver lesions [91].

In a study of 44 patients with hypoenhanced splenic lesions in the parenchymal phase and US-guided sampling with an 18 G needle, bleeding events occurred in 2 patients (4.5%), which were managed conservatively [3]. Specifically, smaller cohort studies are not directly comparable to large meta-analyses because they use different needle gauges or different imaging methods (ultrasound, CT). However, the results regarding diagnostic yield and risks depend on the needle gauge, the access site, the patient’s clinical condition, and the examiner’s experience.

**Figure 17:**
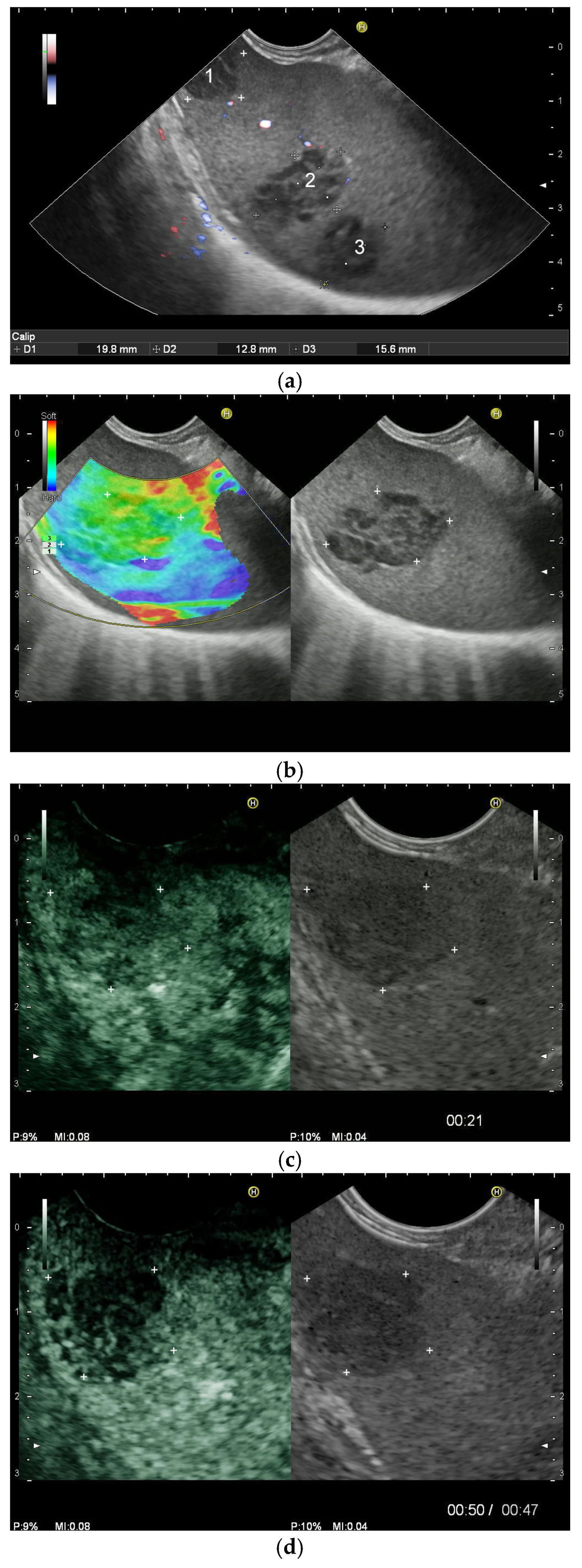

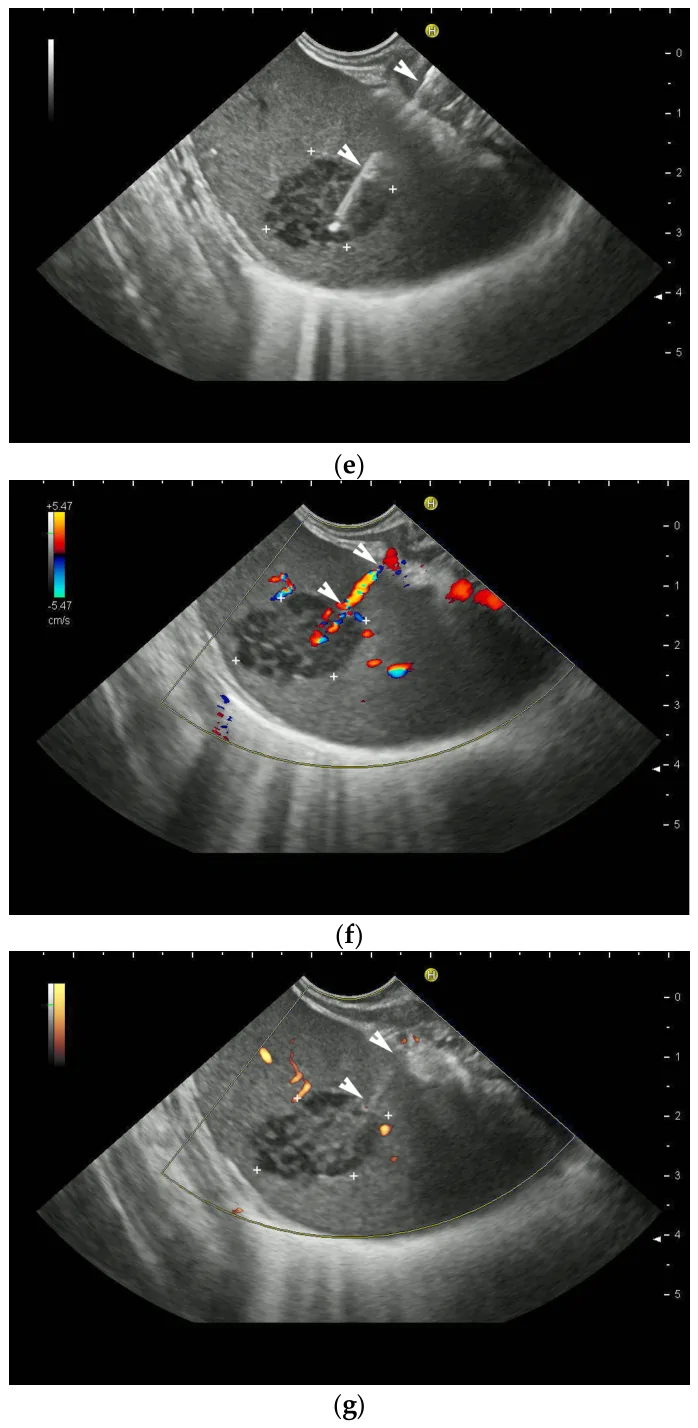
Three hypoechoic and inhomogeneous focal splenic lesions in a patient with a history of breast cancer and a newly discovered lung tumor ((**a**); 1–3, between markers). Strain elastography shows iso-elasticity (**b**) and indicates a relatively soft lesion. After injection of 4.8 mL of the ultrasound contrast agent SonoVue^®^, hypoenhancement of the lesion occurs during the arterial phase (21 s post injection) (**c**), and a rapid and marked washout can be observed as early as the end of the arterial phase ((**d**); 47 s post injection), indicating malignancy. To differentiate between the two possible primary tumors, endoscopic ultrasound-guided fine-needle aspiration was performed ((**e**); the 22 G needle is marked with arrowheads), which allowed for the histological diagnosis of small-cell lung carcinoma. Immediately after the puncture was completed, blood flow along the needle tract (marked with PDF arrowheads) could be observed on color Doppler imaging ((**f**), patent track sign), which stopped just 2 min later, and ((**g**), power Doppler).

For EUS-guided sampling of splenic lesions, there are only smaller series and case reports available that include on results using 25G–19G aspiration and fine-needle biopsy [92,93,94,95,96,97,98,99,100,101,102,103,104]. Adequate specimens were in 92.8% of 62 cases from 6 cohorts of >5 patients; diagnostic accuracy was 88.2%; and only 1 major complication has been reported so far [100]. A “patent track” sign is a phenomenon frequently observed temporarily via color Doppler imaging following EUS-guided splenic sampling, indicating self-limiting bleeding along the needle path [Figure 17]. Therefore, EUS-guided sampling may be regarded as a useful alternative to percutaneous biopsy, especially for small lesions and lesions near the splenic hilum or peripherally near the gastric wall. The advantages of EUS-guided sampling may be a shorter and safer needle path to some focal lesions and better access in case of lung emphysema [93]. There are no direct comparative studies on US- or EUS-guided access, and the choice of access method depends on the location of the lesion, accessibility, clinical indication, and local expertise.

## 8. How Should We Treat Incidentally Discovered Splenic Lesions?

In cases of incidentally diagnosed lesions, it is important to determine whether the lesion could be related to the initial indication for the ultrasound examination [105,106]. Patient age; clinical symptoms such as fever, left flank pain, and B-symptoms; and any known or suspected underlying hematological or oncological disease must be considered. If contrast-enhanced ultrasound (CEUS) shows predominantly benign lesions, follow-up or confirmation with MRI may be performed. If malignancy with progressive washout is suspected, MRI may be performed initially. In cases of concomitant lymphadenopathy (sarcoidosis and lymphomas), histological classification in most can be achieved via lymph node biopsy at a lower risk of adverse events. If the lesion remains unclear and malignancy is neither ruled out nor suspected, histological confirmation of splenic lesions can be obtained through either ultrasound- or EUS-guided sampling. Splenectomy should be an exception in cases where criteria for malignancy exist, and imaging and needle biopsy have not provided a definitive diagnosis.

The American College of Radiologists (ACR) recommends that incidentally discovered splenic lesions with a diameter greater than 1 cm and without clear signs of benignity should be either monitored or further investigated using MRI, PET-CT, or even biopsy [107]. According to the ACR recommendations, MRI should be the preferred method for monitoring. Contrast-enhanced ultrasound (CEUS) is not explicitly mentioned in these recommendations. However, CEUS can be crucial as an initial diagnostic tool in the triage of lesions.

According to guidelines of the European Federation of Societies in Ultrasound in Medicine and Biology (EFSUMB), lesions with low-grade arterial contrast enhancement and progressive contrast washout in the parenchymal phase generally require further imaging or biopsy, particularly in high-risk groups [13,108]. Focal splenic lesions with benign contrast enhancement characteristics are generally suitable for interval imaging [13].

**Figure 18:**
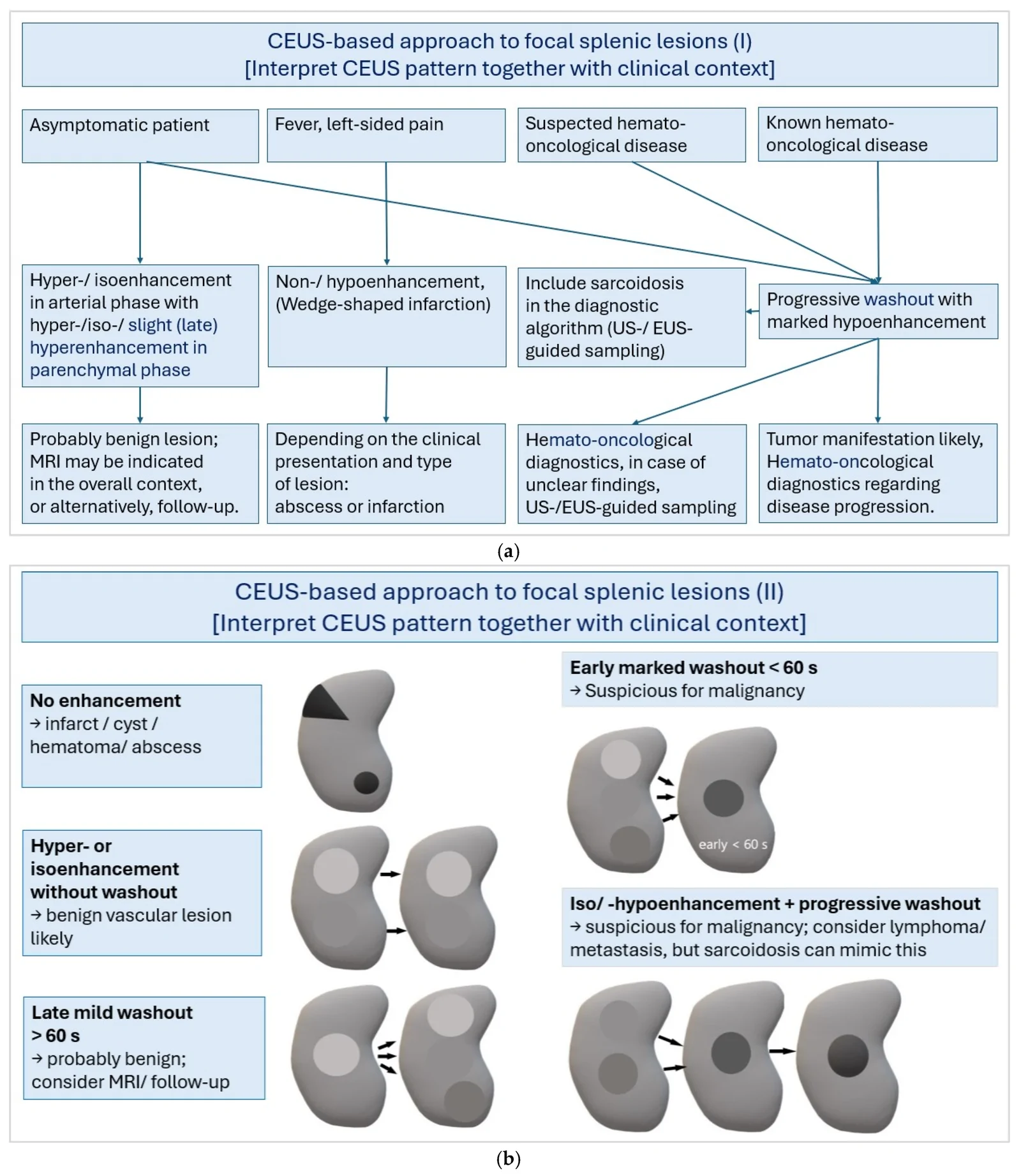
Proposal for a diagnostic algorithm for focal splenic lesions using CEUS. (**a**) illustrates a CEUS-based approach within the clinical context and suggests specific next diagnostic steps. (**b**) demonstrates different enhancement patterns of solid splenic lesions.

Recommendations for the management of incidentally detected splenic lesions are summarized in a position paper of the World Federation for Ultrasound in Medicine and Biology (WFUMB), with CEUS being assigned a key diagnostic role [109]. In Figure 18, we illustrate our algorithm for the diagnosis of focal splenic lesions using CEUS and common enhancement patterns and their significance. A rapid, pronounced parenchymal washout (<60 s) is an important imaging feature associated with malignancy but is not specific for malignant disease. However, it has been repeatedly shown that sarcoidosis and certain benign inflammatory and vascular lesions (SANT, inflammatory pseudotumor, and LCA) exhibit identical early/pronounced hypoenhancement and washout. CEUS findings should therefore be interpreted in conjunction with clinical history, oncological risk, and other imaging findings. In patients with indeterminate lesions, particularly solitary lesions without an established diagnosis, further evaluation by imaging follow-up and/or histological confirmation should be considered according to the individual clinical context.

## 9. Limitations

The rarity of focal splenic lesions is reflected in the limited number of available studies evaluating CEUS of the spleen. Most published studies are retrospective single-center investigations with relatively small patient cohorts. In addition, many studies include a heterogeneous spectrum of lesions, such as cysts, infarctions, traumatic lesions, and abscesses, which are less relevant for the differential diagnosis between benign and malignant solid splenic tumors. Consequently, the number of histologically confirmed solid neoplastic lesions available for analysis is often limited.

Another important limitation is the predominance of case reports and small case series, particularly for rare splenic tumors such as SANT, littoral cell angioma, hemangioendothelioma, and angiosarcoma. For several rare entities, the CEUS characteristics had to be extracted from descriptive tables or figure legends rather than from standardized analyses. Histopathological confirmation was not available for all lesions, especially for benign vascular tumors, where diagnosis was frequently based on imaging findings and follow-up examinations rather than tissue sampling.

Publication bias must also be considered. Given the narrative design and predominance of retrospective case series and case reports, no formal risk-of-bias assessment was performed. Rare or unusual lesions with characteristic imaging findings are more likely to be reported, whereas inconclusive or diagnostically difficult cases may remain unpublished. This may lead to an overrepresentation of typical enhancement patterns and could overestimate the diagnostic performance of CEUS in clinical practice.

A further limitation is the lack of standardized CEUS examination protocols across studies. Differences exist regarding ultrasound contrast agent dosage, timing of arterial and parenchymal phase assessment, duration of follow-up imaging, and interpretation criteria for washout and enhancement intensity. Terminology was also inconsistent between publications, with descriptors of B-mode echogenicity, Doppler vascularization, and CEUS enhancement occasionally used interchangeably, which complicated comparison between studies. Therefore, figures and image descriptions often had to be reviewed carefully to correctly interpret the reported enhancement behavior.

In addition, different ultrasound systems, transducer technologies, contrast-specific imaging modes, and mechanical index settings were used across studies. These technical differences may influence lesion conspicuity and enhancement characteristics and reduce comparability between investigations. Interobserver variability was assessed in only a few studies [26,79], although CEUS interpretation is known to depend heavily on examiner experience and technical expertise.

Finally, most available evidence originates from highly specialized centers with extensive CEUS experience. Therefore, the reported diagnostic performance may not be fully transferable to general clinical practice. High-quality training, standardized examination protocols, and multicenter prospective studies are required to improve reproducibility and establish more robust diagnostic criteria for splenic CEUS.

## 10. Future Perspectives

Future studies should focus on standardized CEUS acquisition and interpretation criteria, prospective multicenter validation, and quantitative assessment of enhancement and washout kinetics. Quantitative CEUS using time–intensity curve analysis may provide a more objective assessment of splenic lesion perfusion. Potential parameters include time to peak, peak enhancement, and wash-in and washout kinetics. However, quantitative measurements are influenced by acquisition protocols, ultrasound equipment, motion correction, and the size and position of the region of interest. Standardization of these factors and prospective multicenter validation are therefore required before quantitative CEUS parameters can be incorporated into a standardized reporting or classification system for focal splenic lesions. Further studies are also warranted to define the role of CEUS-guided biopsy and treatment monitoring.

## 11. Conclusions

CEUS provides clinically relevant information for the characterization of focal splenic lesions by enabling dynamic assessment of arterial and parenchymal enhancement. Across the available literature, benign vascular splenic lesions most commonly demonstrate arterial iso- or hyperenhancement with persistent enhancement or only mild and delayed washout, whereas malignant lesions more frequently exhibit early and progressive washout, resulting in marked hypoenhancement during the parenchymal phase. Early washout, particularly within the first 60 s after contrast administration, appears to be an important imaging feature associated with malignant disease.

However, CEUS enhancement patterns are not pathognomonic, and relevant overlap exists between benign and malignant entities. In particular, sarcoidosis, SANT, littoral cell angioma, and inflammatory pseudotumor may exhibit enhancement patterns that mimic malignant disease. Therefore, CEUS findings should be interpreted in conjunction with clinical history, laboratory findings, and other imaging modalities rather than in isolation. In lesions with indeterminate or suspicious enhancement characteristics, further diagnostic evaluation—including MRI and/or image-guided tissue sampling—should be considered according to the clinical context.

## 12. Take-Home Message

- Benign vascular lesions typically demonstrate hyperenhancement or isoenhancement in the arterial phase with persistent isoenhancement or only slight late hypoenhancement in the parenchymal phase.
- If benign lesions develop washout, it is usually mild and occurs relatively late, generally after 60 s following contrast administration.
- Malignant splenic lesions characteristically show early and progressive washout with marked hypoenhancement in the parenchymal phase.
- Splenic lymphoma manifestations are usually hypoechoic on B-mode ultrasound and most commonly demonstrate hypoenhancement with rapid washout on CEUS.
- Splenic metastases may exhibit variable echogenicity and enhancement patterns in the arterial phase but typically develop rapid washout with marked parenchymal hypoenhancement.
- Lack of enhancement in both the arterial and parenchymal phases is highly suggestive of benign lesions such as infarctions, cysts, hematomas, and many abscesses.

## Figures and Tables

**Table 1:** Classification of splenic lesions.

Benign	
***Vascular***	***Infectious***
Hemangioma	Abscesses (bacterial, fungal)
Hamartoma	Parasites (Echinococcus, Leishmaniasis)
Lymphangioma	***Infarction***
(Littoral cell angioma)	Embolic, EBV infection, hemato-lympho-proliferative disease with splenomegaly
Sclerosing angiomatoid nodular transformation (SANT)	***Traumatic***
***Granulomatous***	Contusion, laceration, hematoma, pseudoaneurysm
Sarcoidosis	***Post inflammatory***
Tuberculosis	Pseudocysts
***Other***	***Congenital cysts***
Extramedullary hematopoiesis	Mesenchymal, epidermoid
**Malignant**	**Variable or unclear biological behavior**
***Primary***	Hemangioendothelioma
Non-Hodgkin lymphoma and subtypes of Hodgkin lymphoma	Hemangiopericytoma
(Hem)angiosarcoma	(Littoral cell angioma)
Histiocytic and dendritic sarcomas	
***Secondary***	
Non-Hodgkin lymphoma, Hodgkin lymphoma	
Metastases	

**Table 3:** Single cases describing ultrasound contrast enhancement patterns in splenic hamartomas.

Study	Patients	B-Mode US	Arterial Phase	Parenchymal Phase	Comments
Von Herbay 2009 [19]	n = 1	Isoechoic	Homogenous-like spleen; eccentric supplying arteries in the periphery, central scar without enhancement.	Homogeneous-like spleen/discreetly hyperenhanced at 60 s; Hyperenhancement at 120–180 s.	
Li 2014 [34]	n = 1	Hypoechoic	Hyperenhancement.	Hyperenhancement.	
Picardi 2022 [4]	n = 1	n.i.	Hyperenhancement.	Hyperenhancement.	Study with splenic nodules in patients with NHL or HL.

n.i. = no information available.

**Table 4:** Littoral cell angioma.

Study	Patients	B-Mode US	Arterial Phase	Parenchymal Phase	Comments
Cui 2013 [41]	n = 1; multiple lesions; splenomegaly	Heterogeneous splenic parenchyma, small hypoechoic lesions up to 30 mm	Hyperenhancement	Hypoenhancement	US-guided sampling
Lerchbaumer 2021 [45]	n = 1, multiple lesions; without splenomegaly	n.i.	“No early enhancement”	Hypoenhancement	US-guided sampling

n.i. = no information available.

**Table 5:** SANT.

Study	Patients	B-Mode US	Arterial Phase	Parenchymal Phase	Comments
Cao 2010 [51]	n = 2; single lesions	Heterogeneous hypoechoic lesion, blood-flow signals in the peripheral area	Homogeneous hyperenhancement at 21 s; isoenhancement at 25 s	Hypoenhancement at 30 s	
		Hypoechoic lesion, branch-shaped and wire-like blood-flow signals inside, and spot-shaped blood-flow signals in the periphery	Hypoenhancement; enhanced in the “branch shaped diffuse pattern” from 12 s p.i.; heterogeneous lobular appearance at 23 s p.i.; enhanced septa within the lesion with washout at 37 s.	Hypoenhancement	
Watanabe 2014 [14]	n = 1; single lesion	Well defined hypoechoic lesion with bright linear echoes in the center accompanied by acoustic shadow, central artery on CDI	Spoke wheel-like enhancement from the center; complete, slightly hypoenhanced at 23 s p.i.	n.i.	UCA Sonazoid was used; At 15 min in the post-vascular phase enhancement persisted in the periphery of the lesion
Menozzi 2015 [55]	n = 1	Isoechoic solid mass, with a thick hyperechoic band with posterior acoustic shadowing in the centre and well-defined margin	Spoke wheel-like vessels from the periphery in the very early arterial phase, homogeneous isoenhancement/slightly hypoenhanced	Slightly hypoenhanced	

s = seconds; p.i. = post injection.

**Table 6:** Further rare benign splenic tumors.

	B-Mode US	Arterial Phase	Parenchymal Phase
Hemophagocytosis Syndrome [19] n = 1	n.i.	Slightly inhomogeneous (only moderately less than in the spleen) with a vascularized rim	Hypoenhancement at 60 s and 120–180 s
Hematolymphangioma [34] n = 2	Solitary hypoechoic	Hyperenhancement (50%)	Hypoenhancement (100%)
	Multiple hypoechoic	Hypoenhancement (50%)	
Inflammatory pseudotumor [34] n = 1	Mixed echogenicity	Hyperenhancement	Hypoenhancement
Extramedullary hemopoiesis [56] n = 1	Isoechoic	Isoenhancement	Inhomogeneous enhancement, central hypoenhancement
Extramedullary hemopoiesis [34] n = 1	Hyperechoic	Isoenhancement	Hypoenhancement

n.i. = no information available.

**Table 7:** Sarcoidosis.

Study	Patients	B-Mode US	Arterial Phase	Parenchymal Phase	Comments
Pérez-Grueso 2007 [60]	n = 1	Splenomegaly, multiple hypoechoic lesions up to 10 mm	Non-enhancing lesions without time specifications or phase assignments		“No hypervascularity” on CDI
Stang 2009 [26]	n = 2; four lesions	Splenomegaly; hypoechoic; <3 cm	Hypoenhancement, homogeneous or heterogeneous	Progressive hypoenhancement, homogeneous or dotted	
Grzelak 2012 [61]	n = 1	Heterogeneous parenchyma, splenomegaly	n.i.	Hypoenhancement, multiple lesions up to 10 mm	The lesions appeared isoechoic on B-mode ultrasound and were only distinguished by hypoenhancement on contrast-enhanced ultrasound
Tana 2013 [59]	n = 1	Splenomegaly, multiple hypoechoic lesions	Slight and more peripherally located enhancement	Rapid and marked washout, marked hypoenhancement	Absence of vascular signals on CDI
Zander 2014 [62]	n = 1	Splenomegaly, multiple hypoechoic lesions, no vessel signals on CDI	Hypoenhancement	Progressive washout with marked hypoenhancement	The histological diagnosis was made by EUS-guided sampling from mediastinal lymph nodes
Picardi 2022 [4]	n = 20	n.i.	Hypoenhancement	Hypoenhancement without later washout	Study with splenic nodules in patients with NHL or HL

**Table 8:** Case studies of non-Hodgkin lymphoma and Hodgkin lymphoma.

Study	Patients	B-Mode US	Arterial Phase	Parenchymal Phase	Comments
Ballestri 2001 [65]	n = 1 (Primary splenic lymphoma)	Anechoic lesions with typical cystic criteria	Isoenhancement	Hypoenhancement	
Stang 2009 [26]	n = 67	Predominantly hypoechoic, rarely iso- or hyperechoic	Isoenhancement 8/67 Hypoenhancement 59/67	Hypoenhancement 100%	Progressive hypoenhancement
Von Herbay 2009 [19]	n = 8	n.i.	Hyperenhancement n = 1/8; Isoenhancement n = 3/8; Inhomogeneous slightly less than spleen n = 2/8; Hypo-/no enhance-ment n = 2/8	*At 60 s:* Inhomogeneous slightly less than spleen n = 2/8; Hypo-/no enhancement n = 6/8 *At 120–180 s*: Hypo-/no enhancement n = 8/8	Small rim in AP n = 2/8
Sutherland 2010 [66]	n = 1 (Primary splenic lymphoma)	Well-defined, homogeneous hypoechoic	Homogeneous isoenhancement	Hypoenhancement	Rapid washout with hypoenhancement at 60 s
Görg 2011 [67]	n = 41	Focal hypoechoic lesions	Isoenhancement n = 19/41 Hypoenhancement n = 22/41	Hypoenhancement 100%	
	n = 10	Diffuse small hypoechoic lesions <5 mm	Isoenhancement 100%	Isoenhancement 100%	B-Mode US was carried out with frequencies up to 6 MHz whereas CEUS was carried out using a low mechanical index and a frequency of 1.5 MHz
Chiavaroli 2011 [33]	n = 1	n.i.	Hypoenhancement	Hypoenhancement	
Yu 2012 [29]	n = 7	Hypoechoic	Hyperenhancement 3/7 Hypoenhancement 4/7	Hypoenhancement 100%	100% inhomogeneous enhancement in the arterial phase. 100% with rapid wash-in in AP and rapid washout in early PV
Li 2014 [34]	n = 8	Diffuse 87.5%, solitary 12.5; Hypoechoic 62.5% Mixed echogenicity 37.5%	Hyperenhancement (50%) Isoenhancement (12.5%) Hypoenhancement (37.5%)	Hypoenhancement 100%	
			Heterogeneous (50%) Rim-like (37.5%) Homogeneous (12.5%)		
Zavariz 2017 [68]	Hairy-cell leukemia n = 1	Hypoechoic, solitary	Hypoenhancement	Hypoenhancement	
D’Auria [69] 2021	n = 1	Multiple, hypoechoic	Isoenhancement	Hypoenhancement	Rapid washout with marked hypoenhancement
Picardi [4] 2022	n = 77 patients, 194 lymphoma nodules (aggressive NHL, classical Hodgkin-Lymphoma, indolent NHL)	n.i.	Isoenhancement 100%	Hypoenhancement 100%	Washout started before 60 s, marked washout and hypoenhancement before 120 s
Zhang 2023 [64]	n = 1; case report (DLBCL)	Hypoechoic	Hypoenhancement, centripetal	Rapid marked washout with hypoenhancement	
Findeisen 2024 [70]	n = 32; Hodgkin-lymphoma	Hypoechoic	Hypo- or isoenhancement	Hypoenhancement	The primary objective of the study was to evaluate the accuracy of CEUS in detecting splenic infiltration in Hodgkin’s disease

CEUS = contrast-enhanced ultrasound; s = seconds, AP = arterial phase; PP = parenchymal phase.

**Table 9:** Enhancement patterns of splenic metastases on CEUS.

Study	B-Mode US	Appearance on CEUS	Arterial Phase	Parenchymal Phase
Von Herbay 2009 [19]; n = 6	n.i.	Hyperenhancement (small rim n = 1/6)	1/6	0/6
		Isoenhancement	1/6	0/6
		Slight hypoenhancement	2/6	At 60 s 1/6
		Hypoenhancement/no enhancement	2/6	At 60 s: 5/6 At 120–180 s: 6/6
Stang 2009 [26]; n = 12	Variable echogenicity, predominantly hypoechoic, solid or cystic–solid, some with a halo-like rim	Progressive hypoenhancement	100%; homogeneous, heterogeneous; rim-like	100% Heterogeneous, rim-like, dotted
Neesse 2010 [1]; n = 32	Hypoechoic 44% Isoechoic 38% Hyperechoic 18%	Hyperenhancement	3%	0%
		Isoenhancement	6%	0%
		Hypoenhancement	66%	75%
		Complex (with rim enhancement and/or necroses)	25%	25%
Yu 2012 [29]; n = 10	Hyperechoic 80% Hypoechoic 10% Mixed 10%	Hyperenhancement	10%	10%
		Isoenhancement	20%	0%
		Hypoenhancement	70%	90%
Li 2014 [34] n = 2	Multiple, hypoechoic n = 1	Hypoenhancement	n = 1	100%
	Solitary, hypoechoic n = 1	Hyperenhancement	n = 1	
Chen 2023 [74] n = 1	Solitary, mixed cystic–solid		Isoenhancement of solid parts	Hypoenhancement of solid parts

n.i. = no information available; s = seconds.

**Table 10:** Studies describing B-Mode and CEUS characteristics to differentiate benign from malignant lesions.

Study	B-Mode	Arterial Phase Enhancement	Parenchymal Phase Enhancement	Benign	Malignant	Comments
Bert 2010 [76]	Hypoechoic	Hyper/iso with centripetal or central filling	Isoenhanced n = 7 Hypoenhanced n = 27 Non-enhanced n = 6 (without further information on B-mode echogenicity and AP enhancement)	100% (n = 15)		Vascular splenic tumor
	Hypoechoic	Hyper/iso with homogeneous filling		Without definitive diagnosis (n = 7)		
	Hyperechoic	Hypoenhanced		Without definitive diagnosis (n = 14)		
	Hypoechoic	Non-enhanced		100% (n = 4)		Cysts
Stang 2011 [38]	Hyperechoic	Hyperenhanced	Isoenhanced	0%	0%	No significant differences
		Hyperenhanced	Hypoenhanced	0%	0%	
		Isoenhanced	Isoenhanced	13%	0%	
		Isoenhanced	Hypoenhanced	33%	18%	
		Hypoenhanced	Hypoenhanced	53%	82%	
	Iso- or hypoechoic	Hyperenhanced	Isoenhanced	11%	0%	
		Hyperenhanced	Hypoenhanced	32%	4%	
		Isoenhanced	Isoenhanced	25%	0%	
		Isoenhanced	Hypoenhanced	11%	18%	
		hypo	hypo	21%	78%	<0.0001
Götzberger 2012 [77]	n.i.	Hyper-/or isoenhanced		90%	0%	
			Rapid washout	7%	100%	
		Non-enhancement		100%	0%	
Yang 2021 [79]	n.i.	Hyperenhancement		38.6%	2.5%	
	n.i.		Hyperenhancement	15.7%	0%	
Safai Zadeh 2024 [2]	Hypoechoic	Hyperenhanced	n.i.	100%	0%	
		Iso-/or hypoenhanced	n.i.	84%	16%	
	Hyperechoic	Hyperenhanced	n.i.	100%	0%	
		Iso-/or hypoenhanced	n.i.	94%	6%	
	Cystic	Non-enhanced	n.i.	100%	0%	

n.i. = no information available; AP = arterial phase.

## Data Availability

No new data were created or analyzed in this study. Data sharing is not applicable to this article.
